# Overexpression of Pyruvate Dehydrogenase Kinase-3 Predicts Poor Prognosis in Urothelial Carcinoma

**DOI:** 10.3389/fonc.2021.749142

**Published:** 2021-09-13

**Authors:** Yu-Hsuan Kuo, Ti-Chun Chan, Hong-Yue Lai, Tzu-Ju Chen, Li-Ching Wu, Chung-Hsi Hsing, Chien-Feng Li

**Affiliations:** ^1^Division of Hematology and Oncology, Department of Internal Medicine, Chi-Mei Medical Center, Tainan, Taiwan; ^2^College of Pharmacy and Science, Chia Nan University, Tainan, Taiwan; ^3^Department of Medical Research, Chi Mei Medical Center, Tainan, Taiwan; ^4^National Institute of Cancer Research, National Health Research Institutes, Tainan, Taiwan; ^5^Department of Clinical Pathology, Chi Mei Medical Center, Tainan, Taiwan; ^6^Department of Anesthesiology, Chi Mei Medical Center, Tainan, Taiwan

**Keywords:** pyruvate dehydrogenase kinase-3, urothelial carcinoma of the bladder, urothelial carcinoma of upper urinary tract, cancer metabolism, metabolic reprogramming

## Abstract

**Background:**

The mitochondrial pyruvate dehydrogenase complex (PDC) link glycolysis to the tricarboxylic acid cycle by decarboxylating pyruvate to acetyl coenzyme A irreversibly. Cancer cells are characterized by a shift in cellular metabolism from mitochondrial respiration to glycolysis. PDC activity inhibition mediated by phosphorylation *via* pyruvate dehydrogenase kinase (PDK) has been linked to cancer. However, the clinical significance of PDKs in urothelial cancer prognosis is not clear. We investigated the role and prognostic value of PDK3 expression in patients with upper urinary tract urothelial carcinoma (UTUC) and urinary bladder urothelial carcinoma (UBUC).

**Patients and Methods:**

We retrospectively analyzed clinical data and pathological features. Formalin-fixed urothelial carcinoma (UC) tissues were collected and embedded in paraffin. The correlation of PDK3 expression with clinical characteristics, pathological findings and patient outcomes, including metastasis-free survival (MFS) and disease-specific survival (DSS) were analyzed by Pearson’s chi-square test, Kaplan–Meier analysis, and the multivariate Cox proportional hazards model.

**Results:**

Data from 295 patients with UBUC and 340 patients with UTUC were evaluated. High PDK3 expression significantly correlated with several pathologic variables such as high T stage, lymph node metastases, high tumor grade, vascular invasion, and high mitotic rate (all *P* < 0.001). High PDK3 expression was associated with poor disease-specific survival (DSS) (*P* < 0.0001) and metastatic free survival (MFS) (*P* < 0.0001) in a Kaplan–Meier analysis. Additionally, multivariate analysis demonstrated increased PDK3 expression is a significant predictive risk factor for DSS [hazard ratio (HR) in UBUC, 2.79, *P* = 0.009; in UTUC, 2.561, *P* = 0.03] and MFS (HR in UBUC, 1.907, *P* = 0.024; in UTUC, 1.793, *P* = 0.044). The gene co-expression analysis showed abundant *PDK3* co-upregulated genes were involved in the processes of DNA replication and repair through the Gene Ontology classification system.

**Conclusion:**

High PDK3 expression has been linked to negative pathologic characteristics and poor oncological outcomes, suggesting that it could be used as a predictive biomarker for UC. PDK3 mRNA levels and its co-upregulated genes were strongly associated with DNA replication and repair. These results suggest that PDK3 may play a key role in tumor proliferation and development.

## Introduction

The most common malignancy of the urinary system is urothelial carcinoma (UC), which includes UC of the urinary bladder (UBUC) and UC of the upper urinary tract (UTUC). Bladder cancer is the 10^th^ most prevalent cancer globally, with about 573,000 new cases and 213,000 deaths in 2020 (1). In Western countries, urinary bladder tumors account for 90–95 percent of UCs, with UTUC accounting for another 5%–10% (2). In Taiwan, UTUC represents as many as 30% of affected cases, and ureteral tumors make up more than half of all UTUC cases (3). UTUC and UBUC are similar in biology and share risk factors; however, they represent different entities due to anatomical and practical differences. Approximately 70% of patients with organ-confined bladder cancer present with a disease confined to the mucosa (stage Ta and CIS) or submucosa (stage T1), referred to as non-muscle-invasive bladder cancer (NMIBC) (4). Despite the good prognosis, patients with low- and intermediate-risk NMIBC have 5-year recurrence-free survival rates of 43% and 33%, respectively, up to 21% of patients with high-risk features develop muscle-invasive bladder cancer (MIBC) (5, 6). In UBUC and UTUC, the gold-standard treatments are transurethral resection of the bladder tumor (TURBT) followed by intravesical chemotherapy and radical nephroureterectomy with bladder cuff excision (7). MIBC accounts for the remaining 30% of localized UC. Neoadjuvant chemotherapy followed by radical cystectomy or bladder preservation strategy with maximum TURBT followed by concomitant chemoradiation are the options for patients with MIBC. The 5-year overall survival rate ranges from 36%-48% (8). Clinical prognostic factors, such as pathological tumor stage, tumor grade, and lymph node metastases define high risk patients. However, a larger, follow-up research is needed to uncover more accurate genomics-based predictors of UC.

Cancer cells have been found to be able to sustain their growth by altering normal metabolic processes. Cancer cells favor aerobic glycolysis over mitochondrial pyruvate oxidation. The mitochondrial pyruvate dehydrogenase complex (PDC) decarboxylates pyruvate to acetyl coenzyme A (acetyl-CoA) irreversibly in normal cells. Pyruvate dehydrogenase kinase has four isoforms (PDK1-4) that can phosphorylate and inhibit the PDC. The affected cells prefer glycolysis over the tricarboxylic acid cycle for adenosine triphosphate (ATP) generation (9). In a variety of cancers, PDK has been linked to tumor aggressiveness, proliferation, and chemotherapy resistance. One study has reported PDK1 expression as an independent prognostic factor that affects overall survival of MIBC patients (10). Another study demonstrated that PDK4 is substantially upregulated in bladder cancer cell lines, and inhibition of PDKs combined with cisplatin therapy could further reduce tumor volume *in vivo* (11). Herein, we assess the genes related to the regulation of acetyl-CoA biosynthetic processes from pyruvate (GO:0010510) using a transcriptome dataset of UC (GSE31684). Among these genes, PDK3 was significantly upregulated in invasive UC compared with non-invasive UC. To date, the possible importance of PDK3 in UC has not been thoroughly investigated. In this work, we looked at PDK3 expression and its prognostic value in the UTUC and UBUC cohorts.

## Patients and Methods

### Data Mining of the Gene Expression Dataset

The NCBI Gene Expression Omnibus (GEO) database yielded a transcriptome dataset (GSE31684), which included 93 UBUC patients who had major operation. We evaluated all probe sets without pre-selection or filtering and loaded the raw files into Nexus Expression 3 software to measure gene expression levels (BioDiscovery, El Segundo, CA, USA). By comparing tumor invasions (high stage vs. low stage) and metastasis (metastasis vs. non-metastasis), comparative studies were undertaken to examine differentially expressed genes associated to the regulation of acetyl-CoA biosynthetic pathway from pyruvate (GO:0010510). For further investigation, the genes expressed differentially (P < 0.05 and log2 ratio >0.3) were chosen.

To examine the roles of PDK3 in UC, the correlations between the PDK3 mRNA level and its co-expressed genes in The Cancer Genome Atlas (TCGA) database were examined using the cBioPortal online platform (http://cbioportal.org). The top 200 genes with a positive correlation or a negative correlation with PDK3 were further investigated using the Gene Ontology (GO) classification system (http://geneontology.org/) in agreement with biological processes or cellular components and ranked by fold enrichment for functional annotation.

### Study Population

Between 1996 and 2004, the Chi Mei Medical Center enrolled 340 patients with UTUC and 295 patients with UBUC who had curative surgery. The institutional review board examined and approved this study (105–01–005). All of the subjects gave their informed consent. Retrospective data on demographics and clinical information was recorded. Patients who had received preoperative chemotherapy or radiotherapy, had other malignancies, or had missing clinical data were excluded. The tumor stage was determined using the system developed by the American Joint Committee on Cancer (AJCC) in 2002. Using the seventh edition of the AJCC staging system, two pathologists assessed tumor specimens and categorized them as low- or high-grade. Cisplatin-based adjuvant treatment was given to all UBUC patients with pT3 or pT4 cancers or lymph nodes metastases.

### Immunohistochemistry and Scoring

The formalin-fixed paraffin embedded samples from the study cohort were cut onto slides and prepared according to standard procedure. After antigen retrieval, sections were incubated with a primary antibody targeting PDK1 (Rabbit polyclonal, 10026-1-AP, 1:100, Proteintech) and PDK3 (Rabbit polyclonal, 12215-1-AP, 1:100, Proteintech), respectively, for 1 hour. The DAKO ChemMate EnVision Kit was used to detect primary antibodies (K5001, Carpinteria, CA, USA). Positive immunoreactivity was confirmed by the presence of brown chromogen in the cytoplasm of tumor cells. As positive controls, cell blocks made from cell lines known to express PDK1 and PDK3 were employed. As a negative control, a sample was incubated without the main antibody. Two pathologists used the H-score to estimate PDK1 and PDK3 immunoreactivity, which was calculated using the following equation: H-score = ΣPi (i + 1), where Pi is the percentage of stained tumor cells of various intensities ranging from 0% to 100%, and i is the staining intensity (0 to 3+). In cases where there were scoring discrepancies, the two pathologists analyzed the slides at the same time and agreed on an H-score. Elevated expression was defined as tumors with H-scores equal to or greater than the median of all scored cases.

### Statistical Analysis

Pearson’s chi-square test was used to analyze the relationship between PDK3 expression and several clinicopathological characteristics. The researchers looked at two end points: metastasis-free survival (MFS) and disease-specific survival (DSS).

The DSS was measured from curative surgery to the time of cancer mortality and the MSS was measured from curative surgery to the first metastasis. Relevant PDK3 expression and clinicopathological features were identified using univariate and multivariate analysis. The Kaplan–Meier method with a log-rank test was applied to create survival curves. To find the independent variables, all significant parameters from the univariate analysis were incorporated in the multivariate Cox proportional hazards model. For statistical analysis, SPSS Statistics V.17.0 software (IBM Armonk, NY, USA) was used. The threshold for statistical significance was set at P < 0.05.

## Results

### Upregulation of PDK3 Gene Links to Metabolic Reprogramming in the UBUC Transcriptome

**Figure 1:**
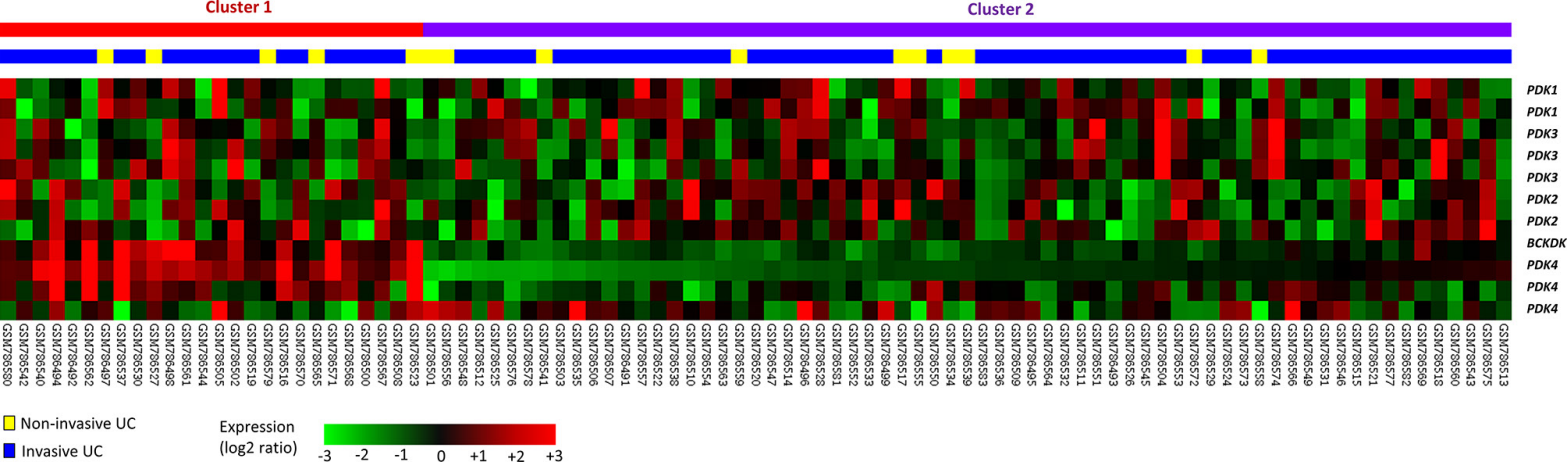
Gene expression patterns from a published transcriptome of urothelial carcinoma (GSE31684) in the Gene Expression Omnibus database connected with the regulation of acetyl-CoA biosynthetic process from pyruvate (GO:0010510). One of the most strongly elevated genes was discovered to be PDK3.

For data mining, we used a published UBUC transcriptome dataset (GSE31684) that includes 93 patients who had undergone radical cystectomy. A total of 78 patients were classified as having invasive disease (pT2–pT4), whereas 15 were classified as having noninvasive or superficial disease (pTa and pT1). We found 11 probes encompassing 5 transcripts linked to the regulation of acetyl-CoA biosynthetic process from pyruvate (GO:0010510). When invasive UC was compared to non-invasive UC, PDK3 was shown to be considerably upregulated (**Table 1** and **Figure 1**). **Table 1** reveals upregulation of the PDK3 gene (Probe: 206348_s_at) in advanced UC with up to 0.4925-fold log ratios (P=0.0235). The goal was to find the most upregulated gene linked to tumor invasiveness, hence PDK3 was selected as a candidate for future testing. The PDK1 gene was found to be nonsignificant after analysis.

**Table 1:** Differential expression of genes related with the regulation of the acetyl-CoA biosynthetic process from pyruvate (GO:0010510) in the transcriptome of urothelial carcinoma of the urinary bladder (GSE31684) in relation to cancer invasiveness.

Probe	Comparing invasive UC to non-invasive		Gene symbol	Gene title	Molecular function
	log ratio	*P*-value			
202030_at	0.3824	0.0666	*BCKDK*	branched chain ketoacid dehydrogenase kinase	ATP binding, kinase activity, protein binding, protein histidine kinase activity, protein kinase activity, protein serine/threonine kinase activity, transferase activity, transferase activity; transferring phosphorus-containing groups
206686_at	-0.0323	0.8638	*PDK1*	pyruvate dehydrogenase kinase; isozyme 1	ATP binding, [pyruvate dehydrogenase (lipoamide)] kinase activity, kinase activity, protein histidine kinase activity, protein kinase activity, transferase activity
226452_at	1.079	0.0748			
202590_s_at	0.2467	0.4089	*PDK2*	pyruvate dehydrogenase kinase; isozyme 2	ATP binding, [pyruvate dehydrogenase (lipoamide)] kinase activity, kinase activity, protein binding, protein histidine kinase activity, protein kinase activity, transferase activity, transferase activity; transferring phosphorus-containing groups
213724_s_at	0.1935	0.0657			
206347_at	0.1944	0.1394	*PDK3*	pyruvate dehydrogenase kinase; isozyme 3	ATP binding, [pyruvate dehydrogenase (lipoamide)] kinase activity, kinase activity, protein histidine kinase activity, protein kinase activity, transferase activity, transferase activity; transferring phosphorus-containing groups
206348_s_at	0.4925	0.0235			
221957_at	0.1477	0.447			
1562321_at	-0.196	0.1584	*PDK4*	pyruvate dehydrogenase kinase; isozyme 4	ATP binding, [pyruvate dehydrogenase (lipoamide)] kinase activity, kinase activity, protein histidine kinase activity, protein kinase activity, transferase activity, transferase activity; transferring phosphorus-containing groups
205960_at	-0.1989	0.1943			
225207_at	-0.1742	0.8394			

### PDK3 Upregulation Is Linked to DNA Replication and Repair

A gene co-expression analysis was undertaken to better understand the role of PDK3 in UC. The top 200 genes from the TCGA database (n = 294) that were correlated positively (**Supplementary Table 1**) or correlated negatively (**Supplementary Table 2**) with PDK3 were examined. We found that, in terms of biological processes (**Supplementary Figure 1**) and cellular components (**Supplementary Figure 2**), abundant PDK3 co-upregulated genes were related to the processes of DNA replication through the GO classification system. The top terms associated with PDK3 upregulation comprised pre-replicative complex assembly involved in cell cycle DNA replication (GO: 1902299, fold enrichment: 49.75), DNA replication, synthesis of RNA primer (GO: 0006269, fold enrichment: 49.75), and DNA replication-dependent nucleosome assembly (GO: 0006335, fold enrichment: 39.8). With respect to cellular components, the top terms associated with PDK3 upregulation contained chromatin assembly factor 1 (CAF-1) complex (GO: 0033186, fold enrichment: 66.33), Elg1 replication factor C (RFC)-like complex (GO: 0031391, fold enrichment: 66.33), alpha DNA polymerase: primase complex (GO: 0005658, fold enrichment: 59.7), CMG complex (GO: 0071162, fold enrichment: 49.75), minichromosome maintenance (MCM) complex (GO: 0042555, fold enrichment: 44.22), DNA replication preinitiation complex (GO: 0031261, fold enrichment: 41.46), and DNA polymerase complex (GO: 0042575, fold enrichment: 31.42). In addition, the biological process terms related to DNA repair, included double-strand break repair *via* break-induced replication (GO: 0000727, fold enrichment: 33.16), nucleotide-excision repair, DNA gap filling (GO: 0006297, fold enrichment: 25.95), and base-excision repair, gap-filling (GO: 0006287, fold enrichment: 17.06), were also associated with PDK3 upregulation. Collectively, the results revealed that the mRNA level of PDK3 and its co-upregulated genes are strongly correlated with DNA replication and repair, suggesting that PDK3 might be important in tumor proliferation and development.

In contrast, regarding the cellular components (**Supplementary Figure 2**), we identified the eukaryotic translation elongation factor 1 complex (GO: 0005853, fold enrichment: 82.16) as the most significantly associated with PDK3 downregulation and identified the eukaryotic translation elongation factor 1 alpha 1 (EEF1A1) gene (Spearman’s correlation: −0.23) to be related to this cellular component. In addition to its function in translation elongation, EEF1A1 has been reported to function as a pro-apoptotic factor (12). Furthermore, we observed that the transcripts of The Gene Expression Profiling Interactive Analysis database (http://gepia.cancer-pku.cn/detail.php?gene=EEF1A1) found that EEF1A1 levels in bladder tumor samples were lower than in matching normal tissues. However, the correlations among the levels of PDK3 and EEF1A1, cell apoptosis, and cancer progression in UC require further analysis.

### Clinicopathological Features of Our UC Cohorts

A total of 340 UTUC and 295 UBUC patients were included in the study (**Table 2**). The median age was 65.8-year-old. In the UTUC cohort, 159 (46.8%) patients had high stage (pT2-4) tumors and 284 patients (83.5%) had high grade tumors. At the time of diagnosis, 28 patients (8.2%) had lymph nodes metastases. Vascular invasion (VI) and perineural invasion (PNI) were found in 106 (31.2%) and 19 (5.9%) of the patients, respectively. Furthermore, 167 lesions (49.1%) demonstrated a high mitotic rate.

**Table 2:** Correlations between PDK3 expression and other clinicopathological parameters in urothelial carcinomas.

Parameter	Category	Upper urinary tract urothelial carcinoma				Urinary bladder urothelial carcinoma			
		Case No.	PDK3 Expression		*P*-value	Case No.	PDK3 Expression		*P*-value
			Low	High			Low	High	
Gender	Male	158	79	79	1.000	216	107	109	0.868
	Female	182	91	91		79	40	39
Age (years)	< 65	138	76	62	0.122	121	67	54	0.112
	≥ 65	202	94	108		174	80	94
Tumor location	Renal pelvis	141	59	82	0.009*	–	–	–	- - -
	Ureter	150	89	61		–	–	–
	Renal pelvis & ureter	49	22	27		–	–	–
Multifocality	Single	278	141	137	0.574	–	–	–	- -
	Multifocal	62	29	33		–	–	–
Primary tumor (T)	Ta	89	69	20	<0.001*	84	64	20	<0.001*
	T1	92	50	42		88	42	46
	T2-T4	159	51	108		123	41	82
Nodal metastasis	Negative (N0)	312	162	150	0.101	266	142	124	<0.001*
	Positive (N1-N2)	28	8	20		29	5	24
Histological grade	Low grade	56	42	14	0.018*	56	42	14	<0.001*
	High grade	284	128	156		239	105	134
Vascular invasion	Absent	234	139	95	<0.001*	246	139	107	<0.001*
	Present	106	31	75		49	8	41
Perineural invasion	Absent	321	163	158	0.238	275	144	131	0.001
	Present	19	7	12		20	3	17
Mitotic rate (per 10 high power fields)	< 10	173	115	58	<0.001*	139	89	50	<0.001*
	>= 10	167	55	112		156	58	98

*Statistically significant.

123 patients (41.7%) in the UBUC cohort had muscle invasive bladder cancer (pT2- 4). At the time of their initial diagnosis, the majority of patients (81%) had tumors with a high histological grade. Only 29 individuals (7.8%) had lymph node metastases when they were diagnosed. There were 156 lesions with a high mitotic rate (52.9%). Furthermore, VI and PNI were found in 20 (6.8%) and 49 (16.6%) of the tumors, respectively.

### Correlations Between PDK3 Expression and Pathological Features in UC

**Figure 2:**
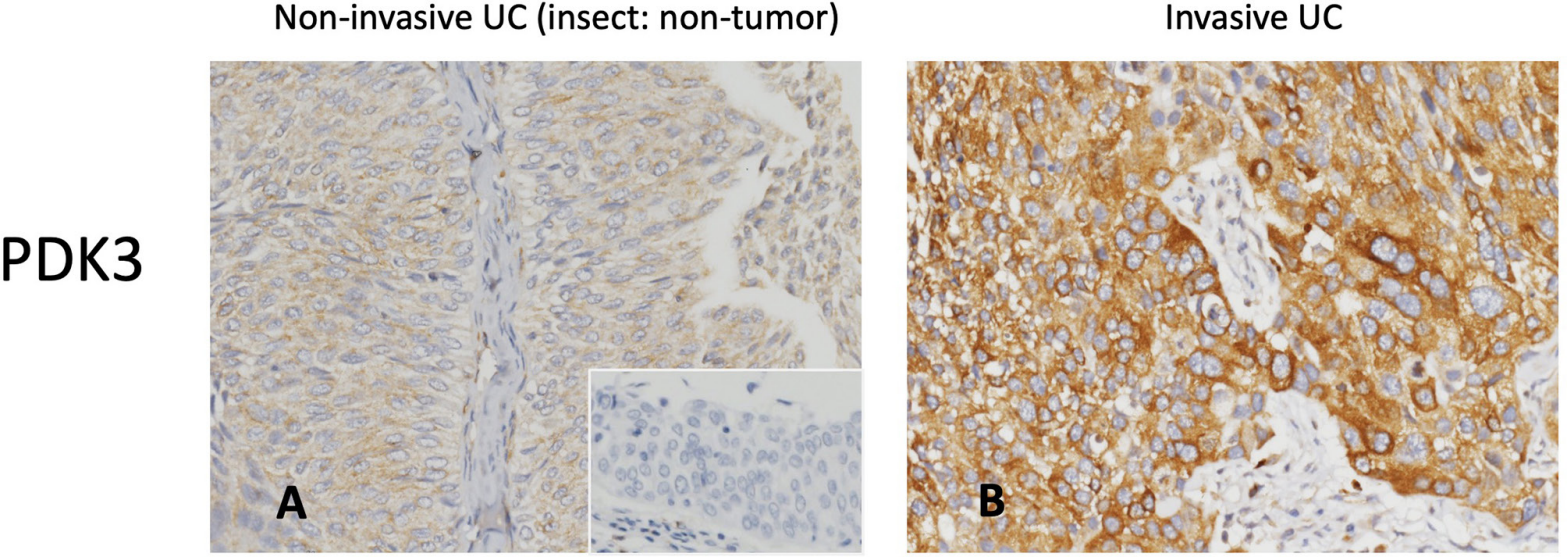
PDK3 immunohistochemistry. Representative sections of non-invasive **(A)** and muscle-invasive **(B)** urothelial carcinoma demonstrate increased PDK3 expression (original magnification: 400×).

Invasive UC had greater PDK3 immunoreactivity than non-invasive UC (**Figure 2**). **Table 2** illustrates the correlation between PDK3 immunoexpression and a variety of clinicopathological factors. In UTUC cohorts, PDK3 overexpression was associated with renal pelvis tumor (*P*=0.009), T2-4 stage (*P <*0.001), high histological grade (*P*=0.018), vascular invasion (*P*<0.001), and high mitotic rate (*P*<0.001). In UBUC cohorts, high PDK3 immunoexpression was associated with T2-4 stage, lymph nodes metastases, high histological grade, vascular invasion, perineural invasion, and high mitotic rate (all *P*<0.001).

### Prognostic Significance of PDK3 Expression

There was a total of 113 patients who died of UC, and 146 patients had subsequent tumor metastases. To investigate the predictive influence of PDK3 expression on cancer metastasis and patient survival in UC, we utilized univariate and multivariate analyses. In univariate analysis at UTUC (**Table 3**), tumor location, multi-focal tumors, T stage, lymph node metastases, vascular invasion, high tumor grade, peri-neural invasion, PDK1 overexpression, and PDK3 overexpression were all found to be significantly associated with poor prognostic factors for MFS and DSS. Multivariate Cox regression analysis showed that lymph node metastases and perineural invasion were significant indicators for DFS, while multi-focal tumors, lymph nodes metastases, vascular invasion, and perineural invasion were significant indicators for MFS. PDK3 expression was significantly associated with poor DFS [hazard ratio (HR), 2.561; 95% confidence interval (CI), 1.070–3.934; *P* = 0.03] and MFS [hazard ratio (HR), 1.793; 95% confidence interval (CI), 1.017–3.163; *P* = 0.044]. In UBUC cohorts (**Table 4**), T stage, lymph nodes metastases, high tumor grade, vascular invasion, peri-neural invasion, high mitotic rate, PDK1, and PDK3 overexpression were significantly associated with poor prognostic factors for DSS and MFS in univariate analysis. T stage, vascular invasion, perineural invasion, and PDK3 overexpression [hazard ratio (HR), 2.790; 95% confidence interval (CI), 1.294–6.016; *P* = 0.009] was significantly associated with DSS in multivariate cox regression analysis. Only T stage and PDK3 overexpression [hazard ratio (HR), 1.907; 95% confidence interval (CI), 1.088–3.341; *P* = 0.024] were significantly associated with MFS.

**Table 3:** Univariate log-rank and multivariate analyses for disease-specific and metastasis-free survival in upper urinary tract urothelial carcinoma.

Parameter	Category	Case No.	Disease-specific Survival					Metastasis-free Survival				
			Univariate analysis		Multivariate analysis			Univariate analysis		Multivariate analysis		
			No. of event	*P*-value	R.R.	95% C.I.	*P*-value	No. of event	*P*-value	R.R.	95% C.I.	*P*-value
Gender	Male	158	28	0.8286	–	–	- -	32	0.7904	–	–	–
	Female	182	33		–	–		38		–	–	–
Age (years)	< 65	138	26	0.9943	–	–	- -	30	0.8470	–	–	–
	≥ 65	202	35		–	–		40		–	–	–
Tumor side	Right	177	34	0.7366	–	–	- - -	38	0.3074	–	–	–
	Left	154	26		–	–		32		–	–	–
	Bilateral	9	1		–	–		0		–	–	–
Tumor location	Renal pelvis	141	24	0.0079*	1	–	0.874	31	0.0659	–	–	–
	Ureter	150	22		0.928	0.498-1.728		25		–	–	–
	Renal pelvis & ureter	49	15		1.316	0.363-4.775		14		–	–	–
Multifocality	Single	278	48	0.0026*	1	–	0.212	52	0.0127*	1	-	0.002*
	Multifocal	62	18		2.165	0.644-7.279		18		2.376	1.359-4.155	
Primary tumor (T)	Ta	89	2	<0.0001*	1	–	0.151	4	<0.0001*	1	–	0.365
	T1	92	9		2.755	0.580-13.075		15		2.249	0.721-7.013	
	T2-T4	159	50		4.274	0.923-19.784		51		2.151	0.662-6.989	
Nodal metastasis	Negative (N0)	312	42	<0.0001*	1	-	<0.001*	55	<0.0001*	1	-	0.001*
	Positive (N1-N2)	28	19		5.124	2.724-9.639		15		2.994	1.591-5.632	
Histological grade	Low grade	56	4	0.0215*	1	–	0.053	3	0.0027*	1	–	0.052
	High grade	284	57		3.073	0.986-9.580		67		3.349	0.990-11.325	
Vascular invasion	Absent	234	24	<0.0001*	1	–	0.144	26	<0.0001*	1	-	0.003*
	Present	106	37		1.580	0.855-2.918		44		2.560	1.386-4.729	
Perineural invasion	Absent	321	50	<0.0001*	1	-	<0.001*	61	<0.0001*	1	-	0.004*
	Present	19	11		4.059	1.898-8.679		9		3.089	1.437-6.639	
Mitotic rate (per 10 high power fields)	< 10	173	27	0.167	–	–	- -	30	0.0823	–	-	-
	>= 10	167	34		–	–		40			-	-
PDK1 expression	Low	170	19	0.0011*	1		0.441	26	0.0116*			0.587
	High	170	42		1.246	0.712-2.179		44		1.159	0.689-1.950	
PDK3 expression	Low	170	16	<0.0001*	1	-	0.030*	18	<0.0001*	1	-	0.044*
	High	170	45		2.561	1.070-3.934		52		1.793	1.017-3.163	

*Statistically significant.

**Table 4:** Univariate log-rank and multivariate analyses for disease-specific and metastasis-free survival in urinary bladder urothelial carcinoma.

Parameter	Category	Case No.	Disease-specific Survival					Metastasis-free Survival				
			Univariate analysis		Multivariate analysis			Univariate analysis		Multivariate analysis		
			No. of event	*P*-value	R.R.	95% C.I.	*P*-value	No. of event	*P*-value	R.R.	95% C.I.	*P*-value
Gender	Male	216	41	0.4446	-	-	- -	60	0.2720	-	-	- -
	Female	79	11		-	-		16		-	-	
Age (years)	< 65	121	17	0.1136	-	-	- -	31	0.6875	-	-	- -
	≥ 65	174	35		-	-		45		-	-	
Primary tumor (T)	Ta	84	1	<0.0001*	1	-	<0.001*	4	<0.0001*	1	-	0.007*
	T1	88	9		4.856	0.488-48.362		23		4.705	1.304-16.983	
	T2-T4	123	42		21.590	2.244-207.718		49		7.467	2.059-27.080	
Nodal metastasis	Negative (N0)	266	41	0.0002*	1	-	0.773	61	<0.0001*	1	-	0.070
	Positive (N1-N2)	29	11		1.111	0.542-2.279		15		1.785	0.953-3.343	
Histological grade	Low grade	56	2	0.0013*	1	-	0.780	5	0.0007*	1	-	0.640
	High grade	239	50		0.793	0.155-4.055		71		0.765	0.249-2.348	
Vascular invasion	Absent	246	37	0.0024*	1	-	0.056	54	0.0001*	1	-	0.754
	Present	49	15		0.508	0.253-1.017		22		0.906	0.490-1.678	
Perineural invasion	Absent	275	44	0.0001*	1	-	0.047*	66	0.0007*	1	-	0.198
	Present	20	8		2.325	1.010-5.353		10		1.624	0.776-3.402	
Mitotic rate (per 10 high power fields)	< 10	139	12	<0.0001*	1	-	0.047*	23	<0.0001*	1	-	0.058
	>= 10	156	40		1.992	1.008-3.937		53		1.653	0.983-2.780	
PDK1 expression	Low	147	13	0.0009	1	-	0.232	25	0.0044	1	-	0.879
	High	148	39		1.494	0.774-2.884		51		1.040	0.627-1.725	
PDK3 expression	Low	147	9	<0.0001*	1	-	0.009	19	<0.0001*	1	-	0.024*
	High	148	43		2.790	1.294-6.016		57		1.907	1.088-3.341	

*Statistically significant.

### Survival Analysis in UTUC and UBUC

**Figure 3:**
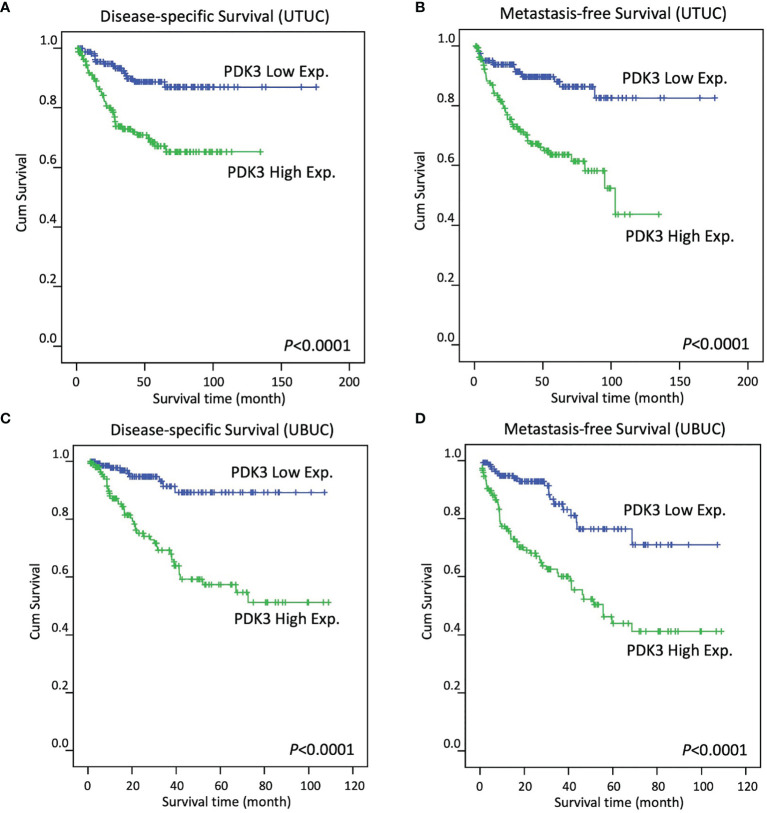
In patients with upper tract urothelial carcinoma (**A, B**, respectively) and urinary bladder urothelial carcinoma (**C, D**, respectively), Kaplan–Meier plots reveal PDK3 overexpression had a significant predictive influence on disease-specific survival and metastasis-free survival.

In UTUC, a significant correlation between increased PDK3 expression and worse DSS (**Figure 3A**; P < 0.0001) and MFS (**Figure 3B**; P < 0.0001) was found by Kaplan–Meier analysis. High PDK3 expression was similarly linked to worse DSS (**Figure 3C**; P <0.0001) and MFS (**Figure 3D**; P < 0.0001) in UBUC cohorts.

## Discussion

Cancer is caused by the uncontrolled expansion of aberrant or mutated cells in the body, and cancer cells are recognized by their ability to quickly divide and proliferate. Cancer cells acquire additional nutrients through multiple mechanisms to meet energy demands. The metabolic characteristics of cancer cells are different from those of normal cells due to their metabolic reprogramming (7). This “metabolic reprogramming” is characterized by their enhanced ability to survive, invade, and metastasize. This concept was originally conceived by Otto Warburg nearly 100 years ago (13). In normal cells, energy production is primarily accomplished *via* mitochondrial oxidative phosphorylation (OXPHOS) and the production of 36 ATPs. Cancer cells, on the other hand, have been found to preferentially use cytoplasmic aerobic glycolysis, which produces two ATPs even when oxygen is present. The mitochondrial pyruvate dehydrogenase complex (PDC) decarboxylates pyruvate to acetyl-CoA, and acts as a bridge between glycolysis and the TCA cycle. PDK1-4 can catalyze PDC phosphorylation, and afflicted cells prefer glycolysis to the tricarboxylic acid cycle for ATP generation (9). Aerobic glycolysis has been found to cause cancer chemoresistance through a variety of mechanisms, including enhanced ATP production and resistance to mitochondrial depolarization, both of which are required for cell death (14). PDK 1–3 functions as an oncogene by interacting with numerous signaling pathways that contribute in cancer growth. PDK4’s function is multifaceted, since it functions as both an oncogene and a tumor suppressor (15). PDK3 has highest affinity to the PDC and is least researched (16). Higher expression of PDK3 is associated with higher tumor stage in many cancers (17). PDK3 knockdown suppressed proliferation and caused apoptosis in gastric cancer cell lines (18) and the prostate cancer cell line (19). Furthermore, higher PDK3 expression has been associated with chemoresistance in gastric cancer cells (20) and colon cancer cells (17). It also has been associated with poor prognosis in cholangiocarcinoma (21), and AML (22). Studies showed mRNA expression of hypoxia inducible factor-1α (HIF-1α) is increased in UC, and it induced glycolytic genes, including PDK-1 and PDK-3 (17, 23). The HIF-1 α genes are involved in many processes such as cellular metabolism, proliferation and survival, angiogenesis, invasion and metastasis of tumor cells (24). It has been known for over a decade that overexpression of HIF-1 α is a common phenomenon in solid tumors and is highly associated with cancer metastases, aggressiveness and chemotherapy resistance (25). This may explain our conclusions that PDK3 expression associated with worse DSS and MFS.

It has recently been discovered that bladder cancer progression is linked to alterations in cell glycolytic characteristics (26) and manifests itself in a more aggressive stage (27). Several studies have found that distinct PDK isozymes are selectively upregulated in bladder cancer. In MIBC, PDK1 expression was linked to a high tumor grade and a high Ki67 index in one study. Another study demonstrated PDK4 is substantially activated in high grade bladder cancer cell lines, and inhibition of PDKs combined with cisplatin therapy could further decrease tumor volume *in vivo* (11). For accuracy, we identified 11 probes covering 5 transcripts (BCKDK, PDK1, PDK2, PDK3, PDK4) associated with the regulation of acetyl-CoA biosynthetic process from pyruvate through transcriptomic profiling. Among these genes, only PDK3 was significantly upregulated in invasive UC compared with non-invasive UC while PDK1 and PDK4 were not. Considering other studies (10) have shown PDK1 expression as an independent prognostic factor, we also performed a survival analysis for PDK1. Kaplan–Meier analysis indicated PDK1 overexpression was a poor prognostic indicator for DSS and MSS in UTUC (**Supplementary Figures 3A, B**) and UBUC, (**Supplementary Figures 3C, D**) respectively. However, it was not significant after multivariate analysis.

For patients with metastatic UC, platinum-based combination chemotherapy is the cornerstone of systemic therapy (28–31). Although response rate can be as high as 50%, it is not durable, as most patients eventually experience disease progression. Since May 2016, five programmed cell death-1 (PD-1) pathway inhibitors have been licensed for usage after platinum-based chemotherapy has failed (32–37). Immunotherapy plays an important role in UC treatment since 2016. Recent work has revealed that immune cells compete for nutrients in the microenvironment with cancer cells and other growing cells (38). Even though many tumor antigens for T cells to identify, the glycolytic activities of cancer cells have been shown to limit glucose intake by Tumor-infiltrating lymphocytes (TIL) T cells, resulting in T-cell depletion and immunological evasion, according to several recent investigations (39, 40). Moreover, glycolytic metabolites, such as high lactate level, low pH and hypoxia are likewise prevalent in the tumor microenvironment and can have a deleterious effect on immune function (41). Emerging evidence indicates glycolysis promotes the PD-L1 expression in tumors, and leads to T-cell inactivation (42). Thus, enhancing tumor immunogenicity by modifying these metabolic pathways could be promising to offset the negative consequences of energy competition between the tumor and the immune cells (43). Combining PDK pathway inhibitors and PD-1 pathway inhibitors may be a promising strategy and worth further investigation. However, because of the various immune cell subtypes and metabolic reprogramming, simultaneously inhibiting tumor cells and activating T-cell function is challenging (44).

Intricate mechanisms are involved in maintaining DNA replication, and dysregulation may cause genome instability and tumor progression. Interestingly, we found many genes in this process co-upregulated with PDK3 (**Supplementary Figures 1** and **2**). When cells enter the G1 phase, a pre-replicative complex (GO: 1902299, fold enrichment: 49.75) is formed at the origin of replication. Upon entering the S phase, the MCM complex (GO: 0042555, fold enrichment: 44.22), GINS complex, and cell division cycle 45 (CDC45) are assembled to form the CMG (CDC45-MCM-GINS) complex (GO: 0071162, fold enrichment: 49.75), which is the active form of the replicative helicase (45). After the helicase unwinds the DNA duplex, the primase (GO: 0005658, fold enrichment: 59.7) generates short RNA primers for DNA synthesis. The ring-shaped sliding clamp, called proliferating cell nuclear antigen (PCNA), is then loaded onto the primer-template junction by the clamp loader/unloader RFC complex (GO: 0031391, fold enrichment: 66.33) (46). Once loaded, the DNA polymerases (GO: 0042575, fold enrichment: 31.42) work in pairs (Pol δ and Pol ϵ) to synthesize DNA with high fidelity. Additionally, during DNA replication, the deposition of newly synthesized histones is mediated by histone chaperones such as CAF-1 (GO: 0033186, fold enrichment: 66.33), which is known as a replication-dependent nucleosome assembly (GO: 0006335, fold enrichment: 39.8) (47). Aberrant DNA replication is leveraged by tumor cells to sustain proliferation. Recently, it has been suggested that DNA replication pathways are enriched in patients with UTUC derived xenografts. It is more prominent with invasive tumors, suggesting that the rate of cell proliferation is critical for UTUC progression (48). However, more research is needed to fully understand the molecular pathways driving PDK3’s proliferative action in UC.

In response to cellular requirements or exogenous stimulation, such as radiation or cytotoxic agents, damaged DNA undergoes repair to maintain normal function and survival. Inhibitors of DNA repair pathways have been shown to be efficacious when combined with DNA-damaging anticancer drugs (49). In addition, numerous DNA replication mediators have been reported to also play a crucial role in DNA repair pathways (50). Impressively, we found that several PDK3 co-upregulated genes that were involved in DNA replication and DNA repair (**Supplementary Figure 1**). The DNA repair pathways included double-strand break repair (GO: 0000727, fold enrichment: 33.16), nucleotide excision repair (GO: 0006297, fold enrichment: 25.95), and base excision repair (GO: 0006287, fold enrichment: 17.06). The genes with functions of both DNA replication and repair are favorable for the cells in order to coordinate these two vital processes promptly and accurately. Targeting these genes may produce a more potent cytotoxic effect for UC therapy. Taken together, the role of PDK3 and glycolysis with DNA replication and repair and the overall contribution to cell survival in UC deserves further investigation.

In conclusion, we found that elevated PDK3 expression was linked to negative pathologic characteristics and poor oncological outcomes, suggesting that it could be used as a prognostic biomarker for UC. PDK3 mRNA levels and its co-upregulated genes are strongly correlated with DNA replication and repair, suggesting that PDK3 may play a key role in tumor proliferation and development. To our knowledge, this is the first study to demonstrate a role of PDK3 in UC. Clarification regarding the detailed mechanism and how to incorporate findings into daily practice needs further investigation.

## Data Availability Statement

The datasets presented in this study can be found in online repositories. The names of the repository/repositories and accession number(s) can be found in the article/**Supplementary Material**.

## Ethics Statement

The studies involving human participants were reviewed and approved by Institutional Review Board (IRB10501-005) of Chi Mei Medical Center. The patients/participants provided their written informed consent to participate in this study.

## Author Contributions

Y-HK, C-HH, and C-FL: study concept and design. T-CC and C-FL: acquisition of data. C-HH, H-YL, and C-FL: analysis and interpretation of data. Y-HK and C-FL: drafting of the manuscript. T-CC, H-YL, and C-FL: statistical analysis. All authors contributed to the article and approved the submitted version.

## Conflict of Interest

The authors declare that the research was conducted in the absence of any commercial or financial relationships that could be construed as a potential conflict of interest.

## Publisher’s Note

All claims expressed in this article are solely those of the authors and do not necessarily represent those of their affiliated organizations, or those of the publisher, the editors and the reviewers. Any product that may be evaluated in this article, or claim that may be made by its manufacturer, is not guaranteed or endorsed by the publisher.

## References

[1] Sung H Ferlay J Siegel RL Laversanne M Soerjomataram I Jemal A. Global Cancer Statistics 2020: GLOBOCAN Estimates of Incidence and Mortality Worldwide for 36 Cancers in 185 Countries. CA Cancer J Clin (2021) 71(3):209–49. doi: 10.3322/caac.21660 33538338

[2] Margulis V Shariat SF Matin SF Kamat AM Zigeuner R Kikuchi E. Outcomes of Radical Nephroureterectomy: A Series From the Upper Tract Urothelial Carcinoma Collaboration. Cancer (2009) 115(6):1224–33. doi: 10.1002/cncr.24135 19156917

[3] Li CC Chang TH Wu WJ Ke HL Huang SP Tsai PC. Significant Predictive Factors for Prognosis of Primary Upper Urinary Tract Cancer After Radical Nephroureterectomy in Taiwanese Patients. Eur Urol (2008) 54(5):1127–34. doi: 10.1016/j.eururo.2008.01.054 18243511

[4] Burger M Catto JW Dalbagni G Grossman HB Herr H Karakiewicz P. Epidemiology and Risk Factors of Urothelial Bladder Cancer. Eur Urol (2013) 63(2):234–41. doi: 10.1016/j.eururo.2012.07.033 22877502

[5] Ritch CR Velasquez MC Kwon D Becerra MF Soodana-Prakash N Atluri VS. Use and Validation of the AUA/SUO Risk Grouping for Nonmuscle Invasive Bladder Cancer in a Contemporary Cohort. J Urol (2020) 203(3):505–11. doi: 10.1097/JU.0000000000000593 31609178

[6] van den Bosch S Alfred Witjes J. Long-Term Cancer-Specific Survival in Patients With High-Risk, Non-Muscle-Invasive Bladder Cancer and Tumour Progression: A Systematic Review. Eur Urol (2011) 60(3):493–500. doi: 10.1016/j.eururo.2011.05.045 21664041

[7] Babjuk M Burger M Comperat EM Gontero P Mostafid AH Palou J. European Association of Urology Guidelines on Non-Muscle-Invasive Bladder Cancer (TaT1 and Carcinoma In Situ) - 2019 Update. Eur Urol (2019) 76(5):639–57. doi: 10.1016/j.eururo.2019.08.016 31443960

[8] Witjes JA Bruins HM Cathomas R Comperat EM Cowan NC Gakis G. European Association of Urology Guidelines on Muscle-Invasive and Metastatic Bladder Cancer: Summary of the 2020 Guidelines. Eur Urol (2021) 79(1):82–104. doi: 10.1016/j.eururo.2020.03.055 32360052

[9] Stacpoole PW. Therapeutic Targeting of the Pyruvate Dehydrogenase Complex/Pyruvate Dehydrogenase Kinase (PDC/PDK) Axis in Cancer. J Natl Cancer Inst (2017) 109(11):djx071. doi: 10.1093/jnci/djx071 29059435

[10] Zhu J Zheng G Xu H Jin X Tang T Wang X. Expression and Prognostic Significance of Pyruvate Dehydrogenase Kinase 1 in Bladder Urothelial Carcinoma. Virchows Arch (2020) 477(5):637–49. doi: 10.1007/s00428-020-02782-z 32388719

[11] Woolbright BL Choudhary D Mikhalyuk A Trammel C Shanmugam S Abbott E. The Role of Pyruvate Dehydrogenase Kinase-4 (PDK4) in Bladder Cancer and Chemoresistance. Mol Cancer Ther (2018) 17(9):2004–12. doi: 10.1158/1535-7163.MCT-18-0063 PMC6724734 29907593

[12] Ruest LB Marcotte R Wang E. Peptide Elongation Factor Eef1a-2/S1 Expression in Cultured Differentiated Myotubes and Its Protective Effect Against Caspase-3-Mediated Apoptosis. J Biol Chem (2002) 277(7):5418–25. doi: 10.1074/jbc.M110685200 PMC2803684 11724805

[13] Pascale RM Calvisi DF Simile MM Feo CF Feo F. The Warburg Effect 97 Years After Its Discovery. Cancers (2020) 12(10):2819. doi: 10.3390/cancers12102819 PMC7599761 33008042

[14] Roh JL Park JY Kim EH Jang HJ Kwon M. Activation of Mitochondrial Oxidation by PDK2 Inhibition Reverses Cisplatin Resistance in Head and Neck Cancer. Cancer Lett (2016) 371(1):20–9. doi: 10.1016/j.canlet.2015.11.023 26607904

[15] Oberhuber M Pecoraro M Rusz M Oberhuber G Wieselberg M Haslinger P. STAT3-Dependent Analysis Reveals PDK4 as Independent Predictor of Recurrence in Prostate Cancer. Mol Syst Biol (2020) 16(4):e9247. doi: 10.15252/msb.20199247 32323921 PMC7178451

[16] Woolbright BL Rajendran G Harris RA Taylor JA 3rd. Metabolic Flexibility in Cancer: Targeting the Pyruvate Dehydrogenase Kinase:Pyruvate Dehydrogenase Axis. Mol Cancer Ther (2019) 18(10):1673–81. doi: 10.1158/1535-7163.MCT-19-0079 31511353

[17] Lu CW Lin SC Chien CW Lin SC Lee CT Lin BW. Overexpression of Pyruvate Dehydrogenase Kinase 3 Increases Drug Resistance and Early Recurrence in Colon Cancer. Am J Pathol (2011) 179(3):1405–14. doi: 10.1016/j.ajpath.2011.05.050 PMC3157210 21763680

[18] Feng L Cheng K Zang R Wang Q Wang J. miR-497-5p Inhibits Gastric Cancer Cell Proliferation and Growth Through Targeting PDK3. Biosci Rep (2019) 39(9):BSR20190654. doi: 10.1042/BSR20190654 31409724 PMC6732365

[19] Wang LY Hung CL Chen YR Yang JC Wang J Campbell M. KDM4A Coactivates E2F1 to Regulate the PDK-Dependent Metabolic Switch Between Mitochondrial Oxidation and Glycolysis. Cell Rep (2016) 16(11):3016–27. doi: 10.1016/j.celrep.2016.08.018 PMC5024724 27626669

[20] Xu J Shi Q Xu W Zhou Q Shi R Ma Y. Metabolic Enzyme PDK3 Forms a Positive Feedback Loop With Transcription Factor HSF1 to Drive Chemoresistance. Theranostics (2019) 9(10):2999–3013. doi: 10.7150/thno.31301 31244938 PMC6568185

[21] Sanmai S Proungvitaya T Limpaiboon T Chua-On D Seubwai W Roytrakul S. Serum Pyruvate Dehydrogenase Kinase as a Prognostic Marker for Cholangiocarcinoma. Oncol Lett (2019) 17(6):5275–82. doi: 10.3892/ol.2019.10185 PMC6507336 31186744

[22] Cui L Cheng Z Liu Y Dai Y Pang Y Jiao Y. Overexpression of PDK2 and PDK3 Reflects Poor Prognosis in Acute Myeloid Leukemia. Cancer Gene Ther (2020) 27(1-2):15–21. doi: 10.1038/s41417-018-0071-9 30578412

[23] Lai Y-W Wu S-B Hsueh TY Chiu AW Wei Y-H Chen SS-S. Enhanced Oxidative Stress and the Glycolytic Switch in Superficial Urothelial Carcinoma of Urinary Bladder. Urological Sci (2016) 27(4):244–9. doi: 10.1016/j.urols.2015.05.004

[24] Soni S Padwad YS. HIF-1 in Cancer Therapy: Two Decade Long Story of a Transcription Factor. Acta Oncol (2017) 56(4):503–15. doi: 10.1080/0284186X.2017.1301680 28358664

[25] Pezzuto A Carico E. Role of HIF-1 in Cancer Progression: Novel Insights. A Review. Curr Mol Med (2018) 18(6):343–51. doi: 10.2174/1566524018666181109121849 30411685

[26] Massari F Ciccarese C Santoni M Iacovelli R Mazzucchelli R Piva F. Metabolic Phenotype of Bladder Cancer. Cancer Treat Rev (2016) 45:46–57. doi: 10.1016/j.ctrv.2016.03.005 26975021

[27] Conde VR Oliveira PF Nunes AR Rocha CS Ramalhosa E Pereira JA. The Progression From a Lower to a Higher Invasive Stage of Bladder Cancer is Associated With Severe Alterations in Glucose and Pyruvate Metabolism. Exp Cell Res (2015) 335(1):91–8. doi: 10.1016/j.yexcr.2015.04.007 25907297

[28] von der Maase H Hansen SW Roberts JT Dogliotti L Oliver T Moore MJ. Gemcitabine and Cisplatin Versus Methotrexate, Vinblastine, Doxorubicin, and Cisplatin in Advanced or Metastatic Bladder Cancer: Results of a Large, Randomized, Multinational, Multicenter, Phase III Study. J Clin Oncol (2000) 18(17):3068–77. doi: 10.1200/JCO.2000.18.17.3068 11001674

[29] Scher HI. A Randomized Comparison of Cisplatin Alone or in Combination With Methotrexate, Vinblastine, and Doxorubicin in Patients With Metastatic Urothelial Carcinoma: A Cooperative Group Study. J Urol (1992) 148(5):1625–6. doi: 10.1200/JCO.1992.10.7.1066 1433578

[30] Logothetis CJ Dexeus FH Finn L Sella A Amato RJ Ayala AG. A Prospective Randomized Trial Comparing MVAC and CISCA Chemotherapy for Patients With Metastatic Urothelial Tumors. J Clin Oncol (1990) 8(6):1050–5. doi: 10.1200/JCO.1990.8.6.1050 2189954

[31] De Santis M Bellmunt J Mead G Kerst JM Leahy M Maroto P. Randomized Phase II/III Trial Assessing Gemcitabine/Carboplatin and Methotrexate/Carboplatin/Vinblastine in Patients With Advanced Urothelial Cancer Who Are Unfit for Cisplatin-Based Chemotherapy: EORTC Study 30986. J Clin Oncol (2012) 30(2):191–9. doi: 10.1200/jco.2011.37.3571 PMC3255563 22162575

[32] Powles T Duran I van der Heijden MS Loriot Y Vogelzang NJ De Giorgi U. Atezolizumab Versus Chemotherapy in Patients With Platinum-Treated Locally Advanced or Metastatic Urothelial Carcinoma (IMvigor211): A Multicentre, Open-Label, Phase 3 Randomised Controlled Trial. Lancet (2018) 391(10122):748–57. doi: 10.1016/S0140-6736(17)33297-X 29268948

[33] Bellmunt J De Wit R Vaughn DJ Fradet Y Lee J-L Fong L. Pembrolizumab as Second-Line Therapy for Advanced Urothelial Carcinoma. New Engl J Med (2017) 376(11):1015–26. doi: 10.1056/nejmoa1613683 PMC5635424 28212060

[34] Patel MR Ellerton J Infante JR Agrawal M Gordon M Aljumaily R. Avelumab in Metastatic Urothelial Carcinoma After Platinum Failure (JAVELIN Solid Tumor): Pooled Results From Two Expansion Cohorts of an Open-Label, Phase 1 Trial. Lancet Oncol (2018) 19(1):51–64. doi: 10.1016/S1470-2045(17)30900-2 29217288 PMC7984727

[35] Sharma P Retz M Siefker-Radtke A Baron A Necchi A Bedke J. Nivolumab in Metastatic Urothelial Carcinoma After Platinum Therapy (CheckMate 275): A Multicentre, Single-Arm, Phase 2 Trial. Lancet Oncol (2017) 18(3):312–22. doi: 10.1016/S1470-2045(17)30065-7 28131785

[36] Sharma P Siefker-Radtke A De Braud F Basso U Calvo E Bono P. Nivolumab Alone and With Ipilimumab in Previously Treated Metastatic Urothelial Carcinoma: CheckMate 032 Nivolumab 1 Mg/Kg Plus Ipilimumab 3 Mg/Kg Expansion Cohort Results. J Clin Oncol (2019) 37(19):1608–16. doi: 10.1200/jco.19.00538 PMC6879315 31100038

[37] Massard C Gordon MS Sharma S Rafii S Wainberg ZA Luke J. Safety and Efficacy of Durvalumab (MEDI4736), an Anti-Programmed Cell Death Ligand-1 Immune Checkpoint Inhibitor, in Patients With Advanced Urothelial Bladder Cancer. J Clin Oncol (2016) 34(26):3119–25. doi: 10.1200/JCO.2016.67.9761 PMC5569690 27269937

[38] Chang C-H Qiu J O’Sullivan D Buck DM Noguchi T Curtis DJ. Metabolic Competition in the Tumor Microenvironment Is a Driver of Cancer Progression. Cell (2015) 162(6):1229–41. doi: 10.1016/j.cell.2015.08.016 PMC4864363 26321679

[39] Qiu J Villa M Sanin DE Buck MD O'Sullivan D Ching R. Acetate Promotes T Cell Effector Function During Glucose Restriction. Cell Rep (2019) 27(7):2063–74.e5. doi: 10.1016/j.celrep.2019.04.022 31091446 PMC6544383

[40] Sukumar M Roychoudhuri R Restifo PN. Nutrient Competition: A New Axis of Tumor Immunosuppression. Cell (2015) 162(6):1206–8. doi: 10.1016/j.cell.2015.08.064 PMC6327313 26359979

[41] Harmon C O'Farrelly C Robinson MW. The Immune Consequences of Lactate in the Tumor Microenvironment. Adv Exp Med Biol (2020) 1259:113–24. doi: 10.1007/978-3-030-43093-1_7 32578174

[42] Jiang Z Liu Z Li M Chen C Wang X. Increased Glycolysis Correlates With Elevated Immune Activity in Tumor Immune Microenvironment. EBioMedicine (2019) 42:431–42. doi: 10.1016/j.ebiom.2019.03.068 PMC6491961 30935888

[43] Kareva I. Metabolism and Gut Microbiota in Cancer Immunoediting, CD8/Treg Ratios, Immune Cell Homeostasis, and Cancer (Immuno)Therapy: Concise Review. Stem Cells (2019) 37(10):1273–80. doi: 10.1002/stem.3051 31260163

[44] Fan C Zhang S Gong Z Li X Xiang B Deng H. Emerging Role of Metabolic Reprogramming in Tumor Immune Evasion and Immunotherapy. Sci China Life Sci (2021) 64(4):534–47. doi: 10.1007/s11427-019-1735-4 32815067

[45] Moyer SE Lewis PW Botchan MR. Isolation of the Cdc45/Mcm2-7/GINS (CMG) Complex, a Candidate for the Eukaryotic DNA Replication Fork Helicase. Proc Natl Acad Sci USA (2006) 103(27):10236–41. doi: 10.1073/pnas.0602400103 PMC1482467 16798881

[46] Boehm EM Gildenberg MS Washington MT. The Many Roles of PCNA in Eukaryotic DNA Replication. Enzymes (2016) 39:231–54. doi: 10.1016/bs.enz.2016.03.003 PMC4890617 27241932

[47] Smith S Stillman B. Purification and Characterization of CAF-I, a Human Cell Factor Required for Chromatin Assembly During DNA Replication In Vitro. Cell (1989) 58(1):15–25. doi: 10.1016/0092-8674(89)90398-x 2546672

[48] Kim K Hu W Audenet F Almassi N Hanrahan AJ Murray K. Modeling Biological and Genetic Diversity in Upper Tract Urothelial Carcinoma With Patient Derived Xenografts. Nat Commun (2020) 11(1):1975. doi: 10.1038/s41467-020-15885-7 32332851 PMC7181640

[49] Helleday T Petermann E Lundin C Hodgson B Sharma RA. DNA Repair Pathways as Targets for Cancer Therapy. Nat Rev Cancer (2008) 8(3):193–204. doi: 10.1038/nrc2342 18256616

[50] Hubscher U. DNA Replication Fork Proteins. Methods Mol Biol (2009) 521:19–33. doi: 10.1007/978-1-60327-815-7_2 19563099

