# Supplementary material for: Overexpression of Pyruvate Dehydrogenase Kinase-3 Predicts Poor Prognosis in Urothelial Carcinoma
Source: Front Oncol. 2021 Sep 13;11:749142. doi: 10.3389/fonc.2021.749142 (PMC8473833; doi:10.3389/fonc.2021.749142)

**Supplementary Table 1. The top 200 genes positively correlated with PDK3.**

| **Correlated Gene** | **Cytoband** | **Spearman's Correlation** | | **p-Value** | **q-Value** |
| --- | --- | --- | --- | --- | --- |
| PIN4 | Xq13.1 | 0.2126551 | 1.47834E-05 | | 0.001103187 |
| DROSHA | 5p13.3 | 0.212664288 | 1.47706E-05 | | 0.001103187 |
| DDX12P | 12p13.31 | 0.212772066 | 1.46205E-05 | | 0.001099232 |
| LINC00634 | 22q13.2 | 0.212786157 | 1.4601E-05 | | 0.001099232 |
| TRIM46 | 1q22 | 0.212989135 | 1.43227E-05 | | 0.001084997 |
| POLD1 | 19q13.3 | 0.213204948 | 1.40323E-05 | | 0.001071113 |
| TFE3 | Xp11.23 | 0.21348429 | 1.36647E-05 | | 0.00104705 |
| MARCKSL1 | 1p35.1 | 0.213671401 | 1.34236E-05 | | 0.001032534 |
| POLE | 12q24.33 | 0.213683415 | 1.34083E-05 | | 0.001032534 |
| SERPINA7 | Xq22.3 | 0.213861427 | 1.31829E-05 | | 0.00102188 |
| C17ORF64 | 17q23.2 | 0.214230173 | 1.27275E-05 | | 0.000990417 |
| CHKA | 11q13.2 | 0.214257116 | 1.26948E-05 | | 0.000990417 |
| FAM199X | Xq22.2 | 0.214482745 | 1.24242E-05 | | 0.000978237 |
| AGO3 | 1p34.3 | 0.215017231 | 1.1805E-05 | | 0.000944351 |
| UXT-AS1 | Xp11.23 | 0.21507969 | 1.17346E-05 | | 0.000942487 |
| CHAF1A | 19p13.3 | 0.215200013 | 1.16E-05 | | 0.000935439 |
| ATAD5 | 17q11.2 | 0.215671315 | 1.1087E-05 | | 0.00090131 |
| SMC1B | 22q13.31 | 0.215733592 | 1.10209E-05 | | 0.00089962 |
| ZNF704 | 8q21.13 | 0.215750471 | 1.1003E-05 | | 0.00089962 |
| ERCC6L | Xq13.1 | 0.215869734 | 1.08776E-05 | | 0.000898931 |
| RPS6KA6 | Xq21.1 | 0.21627832 | 1.04581E-05 | | 0.000867849 |
| ACBD7 | 10p13 | 0.216510399 | 1.02267E-05 | | 0.000852185 |
| MBTPS2 | Xp22.12 | 0.216769598 | 9.97408E-06 | | 0.00083461 |
| XRCC1 | 19q13.31 | 0.216831085 | 9.91503E-06 | | 0.000833154 |
| FOXRED2 | 22q12.3 | 0.216861122 | 9.8863E-06 | | 0.000833154 |
| LIG1 | 19q13.33 | 0.217113254 | 9.64826E-06 | | 0.000817608 |
| BUB1 | 2q13 | 0.21711555 | 9.64611E-06 | | 0.000817608 |
| UHRF1BP1L | 12q23.1 | 0.217293297 | 9.48162E-06 | | 0.000810354 |
| SIX4 | 14q23.1 | 0.217544722 | 9.2535E-06 | | 0.000794252 |
| E2F1 | 20q11.22 | 0.218116833 | 8.75373E-06 | | 0.000754594 |
| OR10H1 | 19p13.12 | 0.218504571 | 8.42974E-06 | | 0.000729811 |
| TMEM86A | 11p15.1 | 0.218514024 | 8.42199E-06 | | 0.000729811 |
| RNFT2 | 12q24.22 | 0.218533813 | 8.40578E-06 | | 0.000729811 |
| POLA2 | 11q13.1 | 0.218644772 | 8.31543E-06 | | 0.000729387 |
| SAP30 | 4q34.1 | 0.218722691 | 8.25254E-06 | | 0.000727059 |
| RTL8B | Xq26.3 | 0.218730818 | 8.246E-06 | | 0.000727059 |
| RORC | 1q21 | 0.219259749 | 7.83122E-06 | | 0.000696073 |
| LAS1L | Xq12 | 0.219485623 | 7.66021E-06 | | 0.000683913 |
| TACC3 | 4p16.3 | 0.219495694 | 7.65267E-06 | | 0.000683913 |
| ENO2 | 12p13.31 | 0.219760724 | 7.45674E-06 | | 0.000675859 |
| NADK2 | 5p13.2 | 0.22043337 | 6.98069E-06 | | 0.000637474 |
| KDM5C | Xp11.22 | 0.220748755 | 6.7676E-06 | | 0.000623711 |
| TDRKH | 1q21.3 | 0.220907773 | 6.66252E-06 | | 0.000619738 |
| ALMS1 | 2p13.1 | 0.220985338 | 6.61183E-06 | | 0.000617897 |
| CEP78 | 9q21.2 | 0.221099478 | 6.53791E-06 | | 0.000613857 |
| PARPBP | 12q23.2 | 0.221232523 | 6.45274E-06 | | 0.000608718 |
| POLE2 | 14q21.3 | 0.221616109 | 6.21305E-06 | | 0.000588886 |
| NUP62 | 19q13.33 | 0.2217435 | 6.13535E-06 | | 0.000587085 |
| RFC4 | 3q27.3 | 0.221897217 | 6.04282E-06 | | 0.000581011 |
| ATP7A | Xq21.1 | 0.222455547 | 5.7178E-06 | | 0.000563666 |
| DNAJC22 | 12q13.12 | 0.222605243 | 5.63354E-06 | | 0.000560523 |
| SEPHS1 | 10p13 | 0.223420785 | 5.19487E-06 | | 0.000530062 |
| HMGB2 | 4q34.1 | 0.223505948 | 5.15098E-06 | | 0.00052828 |
| RPIA | 2p11.2 | 0.223750835 | 5.02677E-06 | | 0.000518197 |
| ZNF674 | Xp11.3 | 0.223820273 | 4.99207E-06 | | 0.000517287 |
| RIBC1 | Xp11.22 | 0.224049092 | 4.87933E-06 | | 0.000508238 |
| PSMD11 | 17q11.2 | 0.224237783 | 4.7882E-06 | | 0.000501357 |
| GINS1 | 20p11.21 | 0.225248077 | 4.32724E-06 | | 0.000455477 |
| HPRT1 | Xq26.2-q26.3 | 0.225374408 | 4.27268E-06 | | 0.000452113 |
| MICB | 6p21.33 | 0.225473705 | 4.23025E-06 | | 0.000451629 |
| KNTC1 | 12q24.31 | 0.22551134 | 4.21427E-06 | | 0.000451629 |
| HUWE1 | Xp11.22 | 0.226158366 | 3.94846E-06 | | 0.00042684 |
| RPS6KA3 | Xp22.12 | 0.227332447 | 3.50649E-06 | | 0.000385309 |
| USP27X | Xp11.23 | 0.22741231 | 3.47821E-06 | | 0.000384313 |
| NXT2 | Xq23 | 0.227636701 | 3.39991E-06 | | 0.000379859 |
| SPC25 | 2q31.1 | 0.22765649 | 3.39308E-06 | | 0.000379859 |
| UBE2A | Xq24 | 0.22815669 | 3.22489E-06 | | 0.000364376 |
| CDKN3 | 14q22.2 | 0.228238496 | 3.19815E-06 | | 0.000364376 |
| TROAP | 12q13.12 | 0.228712546 | 3.04732E-06 | | 0.000350249 |
| PHEX | Xp22.11 | 0.228787678 | 3.02405E-06 | | 0.000349584 |
| CRACD | 4q12 | 0.229208681 | 2.89676E-06 | | 0.000340778 |
| KIF18B | 17q21.31 | 0.229250556 | 2.88438E-06 | | 0.000340778 |
| GALM | 2p22.1 | 0.229759766 | 2.73787E-06 | | 0.00032592 |
| APIP | 11p13 | 0.230152187 | 2.62983E-06 | | 0.000316832 |
| CA5BP1 | Xp22.2 | 0.230416874 | 2.55928E-06 | | 0.000312092 |
| HDAC6 | Xp11.23 | 0.230548672 | 2.52483E-06 | | 0.00030978 |
| NABP2 | 12q13.3 | 0.230700622 | 2.48566E-06 | | 0.000306857 |
| NUSAP1 | 15q14 | 0.230990035 | 2.41266E-06 | | 0.000300952 |
| CDK4 | 12q14.1 | 0.231054525 | 2.39667E-06 | | 0.000300952 |
| RPAP3 | 12q13.11 | 0.231335457 | 2.32821E-06 | | 0.000296573 |
| HTATSF1 | Xq26.3 | 0.231718513 | 2.23787E-06 | | 0.000286893 |
| ZXDB | Xp11.21 | 0.231944947 | 2.18606E-06 | | 0.000282058 |
| MYBL2 | 20q13.12 | 0.232334972 | 2.09949E-06 | | 0.000272648 |
| SELENBP1 | 1q21.3 | 0.232686932 | 2.0242E-06 | | 0.000264587 |
| KCNK5 | 6p21.2 | 0.232913974 | 1.977E-06 | | 0.000260119 |
| SPATS2 | 12q13.12 | 0.233168049 | 1.92544E-06 | | 0.000255012 |
| SALL2 | 14q11.2 | 0.233342262 | 1.89083E-06 | | 0.000252098 |
| MSL3 | Xp22.2 | 0.233409226 | 1.87769E-06 | | 0.000252098 |
| CCHCR1 | 6p21.33 | 0.233626296 | 1.83568E-06 | | 0.000249739 |
| SMC1A | Xp11.22 | 0.233783978 | 1.80573E-06 | | 0.000247348 |
| LIN7A | 12q21.31 | 0.234073362 | 1.75198E-06 | | 0.00024164 |
| EOLA2 | Xq28 | 0.234378882 | 1.69689E-06 | | 0.000237315 |
| CIT | 12q24.23 | 0.234590375 | 1.65973E-06 | | 0.000233753 |
| BEX3 | Xq22.2 | 0.236213418 | 1.39941E-06 | | 0.00020731 |
| C5ORF22 | 5p13.3 | 0.237467892 | 1.2255E-06 | | 0.000184277 |
| TAF9B | Xq21.1 | 0.237759424 | 1.18816E-06 | | 0.000180015 |
| VBP1 | Xq28 | 0.238236655 | 1.12937E-06 | | 0.000173741 |
| SCPEP1 | 17q22 | 0.238906826 | 1.05151E-06 | | 0.000163017 |
| POP4 | 19q12 | 0.23955774 | 9.81E-07 | | 0.000154454 |
| PABIR3 | Xq26.3 | 0.241406432 | 8.04E-07 | | 0.000129682 |
| ZXDA | Xp11.21 | 0.24169178 | 7.80E-07 | | 0.000127645 |
| VRK3 | 19q13.33 | 0.241914914 | 7.61E-07 | | 0.000126841 |
| GTSE1 | 22q13.31 | 0.242045309 | 7.50E-07 | | 0.000126115 |
| CENPA | 2p23.3 | 0.242469533 | 7.17E-07 | | 0.000121472 |
| PHKA1 | Xq13.1 | 0.243240416 | 6.59E-07 | | 0.00011463 |
| OR10H5 | 19p13.12 | 0.243338265 | 6.52E-07 | | 0.000114411 |
| RIBC2 | 22q13.31 | 0.243381278 | 6.49E-07 | | 0.000114411 |
| MPHOSPH9 | 12q24.31 | 0.243817298 | 6.19E-07 | | 0.000111528 |
| TRMT2B | Xq22.1 | 0.243871187 | 6.15E-07 | | 0.000111528 |
| TXNDC16 | 14q22.1 | 0.244116781 | 5.99E-07 | | 0.000109922 |
| CCDC15 | 11q24.2 | 0.244297355 | 5.87E-07 | | 0.000108773 |
| SNAPIN | 1q21.3 | 0.24472635 | 5.60E-07 | | 0.000104757 |
| CENPM | 22q13.2 | 0.245059934 | 5.40E-07 | | 0.00010195 |
| HMGN2 | 1p36.11 | 0.245458009 | 5.17E-07 | | 9.94685E-05 |
| NUP210 | 3p25.1 | 0.245463839 | 5.17E-07 | | 9.94685E-05 |
| CDK2 | 12q13.2 | 0.245756255 | 5.01E-07 | | 9.81483E-05 |
| CDC25C | 5q31.2 | 0.245951317 | 4.90E-07 | | 9.70154E-05 |
| SNRNP27 | 2p13.3 | 0.246269176 | 4.73E-07 | | 9.46143E-05 |
| TCF19 | 6p21.33 | 0.246530319 | 4.60E-07 | | 9.28569E-05 |
| GEMIN2 | 14q21.1 | 0.247364633 | 4.19E-07 | | 8.55399E-05 |
| REXO5 | 16p12.3 | 0.24739891 | 4.18E-07 | | 8.55399E-05 |
| HCCS | Xp22.2 | 0.247511282 | 4.12E-07 | | 8.55399E-05 |
| TRAFD1 | 12q24.13 | 0.247532661 | 4.11E-07 | | 8.55399E-05 |
| STAG2 | Xq25 | 0.247806525 | 3.99E-07 | | 8.49169E-05 |
| ARAF | Xp11.3 | 0.247922962 | 3.94E-07 | | 8.4728E-05 |
| CSTF2 | Xq22.1 | 0.248241528 | 3.80E-07 | | 8.26711E-05 |
| OTUD5 | Xp11.23 | 0.248354254 | 3.76E-07 | | 8.25385E-05 |
| FBXL20 | 17q12 | 0.248814169 | 3.57E-07 | | 7.96768E-05 |
| SRP54 | 14q13.2 | 0.25060895 | 2.92E-07 | | 6.89715E-05 |
| FADS1 | 11q12.2 | 0.250618138 | 2.92E-07 | | 6.89715E-05 |
| KLHL15 | Xp22.11 | 0.250851364 | 2.84E-07 | | 6.89715E-05 |
| NASP | 1p34.1 | 0.253393706 | 2.13E-07 | | 5.32808E-05 |
| MCM2 | 3q21.3 | 0.253812983 | 2.03E-07 | | 5.14429E-05 |
| HAUS5 | 19q13.12 | 0.254006808 | 1.99E-07 | | 5.12809E-05 |
| CAPSL | 5p13.2 | 0.254492408 | 1.88E-07 | | 4.95987E-05 |
| MB | 22q12.3 | 0.254539195 | 1.87E-07 | | 4.95987E-05 |
| GNL3L | Xp11.22 | 0.254649947 | 1.85E-07 | | 4.95987E-05 |
| PDE7A | 8q13.1 | 0.254660371 | 1.84E-07 | | 4.95987E-05 |
| SUV39H1 | Xp11.23 | 0.254857024 | 1.80E-07 | | 4.95987E-05 |
| CHAF1B | 21q22.12-q22.13 | 0.255305984 | 1.71E-07 | | 4.89572E-05 |
| PRDX4 | Xp22.11 | 0.255615185 | 1.65E-07 | | 4.79373E-05 |
| USP9X | Xp11.4 | 0.256409921 | 1.51E-07 | | 4.43977E-05 |
| TLCD4 | 1p21.3 | 0.256890155 | 1.43E-07 | | 4.26353E-05 |
| MCM5 | 22q12.3 | 0.257920944 | 1.27E-07 | | 3.8421E-05 |
| HPS3 | 3q24 | 0.258910742 | 1.13E-07 | | 3.53221E-05 |
| C6ORF223 | 6p21.1 | 0.259091158 | 1.11E-07 | | 3.51375E-05 |
| CDC6 | 17q21.2 | 0.259176655 | 1.10E-07 | | 3.51375E-05 |
| SAE1 | 19q13.32 | 0.259620492 | 1.04E-07 | | 3.41192E-05 |
| MOSPD1 | Xq26.3 | 0.25999648 | 9.96E-08 | | 3.31984E-05 |
| EBP | Xp11.23 | 0.260927972 | 8.93E-08 | | 3.02739E-05 |
| BCLAF3 | Xp22.12 | 0.261389477 | 8.46E-08 | | 2.91719E-05 |
| P2RX4 | 12q24.31 | 0.262676815 | 7.27E-08 | | 2.55066E-05 |
| ATP6AP2 | Xp11.4 | 0.264635738 | 5.76E-08 | | 2.05797E-05 |
| SPAG5 | 17q11.2 | 0.265188943 | 5.39E-08 | | 1.96165E-05 |
| TOP2A | 17q21.2 | 0.267484454 | 4.10E-08 | | 1.54582E-05 |
| PHF6 | Xq26.2 | 0.268024585 | 3.84E-08 | | 1.47617E-05 |
| APOO | Xp22.11 | 0.26878628 | 3.50E-08 | | 1.37267E-05 |
| SYCE2 | 19p13.13 | 0.269057557 | 3.39E-08 | | 1.35484E-05 |
| HELLS | 10q23.33 | 0.269303795 | 3.29E-08 | | 1.3418E-05 |
| PARP2 | 14q11.2 | 0.2694379 | 3.23E-08 | | 1.3418E-05 |
| KIF4A | Xq13.1 | 0.269999056 | 3.02E-08 | | 1.28553E-05 |
| YY2 | Xp22.12 | 0.270237999 | 2.93E-08 | | 1.2758E-05 |
| TMPO-AS1 | 12q23.1 | 0.270481941 | 2.85E-08 | | 1.26594E-05 |
| MORN2 | 2p22.1 | 0.270774534 | 2.75E-08 | | 1.2493E-05 |
| RFC5 | 12q24.23 | 0.273599751 | 1.94E-08 | | 9.11138E-06 |
| ASF1B | 19p13.12 | 0.273697635 | 1.92E-08 | | 9.11138E-06 |
| TMPO | 12q23.1 | 0.273920083 | 1.87E-08 | | 9.11138E-06 |
| MED14 | Xp11.4 | 0.27635712 | 1.38E-08 | | 7.25962E-06 |
| APPL2 | 12q23.3 | 0.276912623 | 1.29E-08 | | 6.95555E-06 |
| TIMELESS | 12q13.3 | 0.277964791 | 1.13E-08 | | 6.26488E-06 |
| SALL4 | 20q13.2 | 0.27886064 | 1.01E-08 | | 5.75644E-06 |
| FUNDC1 | Xp11.3 | 0.279937495 | 8.79E-09 | | 5.17153E-06 |
| PIMREG | 17p13.2 | 0.281284023 | 7.41E-09 | | 4.63084E-06 |
| CXORF38 | Xp11.4 | 0.281655948 | 7.07E-09 | | 4.55889E-06 |
| SCML1 | Xp22.13 | 0.28265087 | 6.22E-09 | | 4.14832E-06 |
| CCDC22 | Xp11.23 | 0.284431693 | 4.95E-09 | | 3.41349E-06 |
| SLC9A7 | Xp11.3 | 0.285429215 | 4.35E-09 | | 3.10782E-06 |
| STRADB | 2q33.1 | 0.28606304 | 4.01E-09 | | 2.96868E-06 |
| LPIN2 | 18p11.31 | 0.28654451 | 3.76E-09 | | 2.89592E-06 |
| GRIPAP1 | Xp11.23 | 0.28808398 | 3.08E-09 | | 2.48676E-06 |
| TMEM106C | 12q13.11 | 0.290630739 | 2.20E-09 | | 1.91596E-06 |
| NONO | Xq13.1 | 0.292459268 | 1.73E-09 | | 1.57173E-06 |
| PRPS2 | Xp22.2 | 0.293151879 | 1.58E-09 | | 1.50139E-06 |
| CENPI | Xq22.1 | 0.293414435 | 1.52E-09 | | 1.50139E-06 |
| DYNLT3 | Xp11.4 | 0.293447122 | 1.52E-09 | | 1.50139E-06 |
| MCM6 | 2q21.3 | 0.294480031 | 1.32E-09 | | 1.46645E-06 |
| CTPS2 | Xp22.2 | 0.296449379 | 1.01E-09 | | 1.19105E-06 |
| PRIM1 | 12q13.3 | 0.300590027 | 5.76E-10 | | 7.20E-07 |
| ELK1 | Xp11.23 | 0.304996235 | 3.13E-10 | | 4.47E-07 |
| UBA1 | Xp11.3 | 0.306872469 | 2.41E-10 | | 3.70E-07 |
| JADE3 | Xp11.3 | 0.318214505 | 4.71E-11 | | 7.86E-08 |
| FANCB | Xp22.2 | 0.330448278 | 7.51E-12 | | 1.37E-08 |
| APEX2 | Xp11.21 | 0.333723869 | 4.53E-12 | | 1.01E-08 |
| SMS | Xp22.11 | 0.336058075 | 3.15E-12 | | 7.87E-09 |
| TSR2 | Xp11.22 | 0.339032415 | 1.97E-12 | | 6.57E-09 |
| CDK16 | Xp11.3 | 0.342902203 | 1.06E-12 | | 4.25E-09 |
| GPKOW | Xp11.23 | 0.350329573 | 3.17E-13 | | 1.59E-09 |
| POLA1 | Xp22.11-p21.3 | 0.360683225 | 5.58E-14 | | 3.72E-10 |
| SYAP1 | Xp22.2 | 0.374901895 | 4.61E-15 | | 4.61E-11 |
| RBBP7 | Xp22.2 | 0.444411777 | 3.53E-21 | | 7.06E-17 |

**Supplementary Table 2. The top 200 genes negatively correlated with PDK3.**

| **Correlated Gene** | **Cytoband** | **Spearman's Correlation** | **p-Value** | **q-Value** |
| --- | --- | --- | --- | --- |
| TNNT3 | 11p15.5 | -0.337722217 | 2.42E-12 | 6.92E-09 |
| FAM83H | 8q24.3 | -0.332557562 | 5.43E-12 | 1.09E-08 |
| IGF2-AS | 11p15.5 | -0.303802225 | 3.70E-10 | 4.93E-07 |
| IGF2 | 11p15.5 | -0.288013129 | 3.11E-09 | 2.48676E-06 |
| PLEKHN1 | 1p36.33 | -0.280144749 | 8.56E-09 | 5.17153E-06 |
| SERPINB2 | 18q21.33-q22.1 | -0.273904641 | 1.87E-08 | 9.11138E-06 |
| H19 | 11p15.5 | -0.273532256 | 1.96E-08 | 9.11138E-06 |
| PERM1 | 1p36.33 | -0.266645017 | 4.53E-08 | 1.67838E-05 |
| SNHG7 | 9q34.3 | -0.258086676 | 1.24E-07 | 3.82702E-05 |
| RIOX2 | 3q11.2 | -0.254472907 | 1.88E-07 | 4.95987E-05 |
| ALS2CL | 3p21.31 | -0.253952565 | 2.00E-07 | 5.12809E-05 |
| KRT13 | 17q21.2 | -0.250724856 | 2.88E-07 | 6.89715E-05 |
| CAST | 5q15 | -0.250571669 | 2.93E-07 | 6.89715E-05 |
| GAS5 | 1q25.1 | -0.250093379 | 3.09E-07 | 7.19263E-05 |
| REEP5 | 5q22.2 | -0.249972525 | 3.14E-07 | 7.20686E-05 |
| PHYHIP | 8p21.3 | -0.249675515 | 3.24E-07 | 7.36577E-05 |
| PERP | 6q23.3 | -0.248770527 | 3.59E-07 | 7.96768E-05 |
| TRAF3IP2 | 6q21 | -0.24535447 | 5.23E-07 | 9.96477E-05 |
| RPL4 | 15q22.31 | -0.243605627 | 6.33E-07 | 0.000113112 |
| NANOS1 | 10q26.11 | -0.242760536 | 6.94E-07 | 0.000119566 |
| PLEC | 8q24.3 | -0.242693748 | 6.99E-07 | 0.000119566 |
| SOX15 | 17p13.1 | -0.241821447 | 7.69E-07 | 0.000127071 |
| KRT24 | 17q21.2 | -0.241628028 | 7.85E-07 | 0.000127645 |
| SREK1IP1 | 5q12.3 | -0.240803909 | 8.58E-07 | 0.000137276 |
| MMS19 | 10q24.1 | -0.239609862 | 9.75E-07 | 0.000154454 |
| TPT1 | 13q14.13 | -0.23896902 | 1.04455E-06 | 0.000163017 |
| RIN1 | 11q13.2 | -0.23796173 | 1.16289E-06 | 0.000177532 |
| LAD1 | 1q32.1 | -0.237159574 | 1.26622E-06 | 0.000188978 |
| CYP2W1 | 7p22.3 | -0.235923667 | 1.44282E-06 | 0.000212169 |
| AHNAK | 11q12.3 | -0.235736364 | 1.47156E-06 | 0.000214816 |
| ZNF276 | 16q24.3 | -0.235649788 | 1.48503E-06 | 0.000215212 |
| ZFAS1 | 20q13.13 | -0.235490416 | 1.51014E-06 | 0.000217275 |
| N4BP1 | 16q12.1 | -0.235171321 | 1.56163E-06 | 0.000223079 |
| PPT2 | 6p21.32 | -0.234701511 | 1.64052E-06 | 0.000232686 |
| PLD1 | 3q26.31 | -0.234089646 | 1.749E-06 | 0.00024164 |
| EEF1A1P9 | 4q24 | -0.233389084 | 1.88163E-06 | 0.000252098 |
| BOK | 2q37.3 | -0.23100523 | 2.40888E-06 | 0.000300952 |
| ESRRA | 11q13.1 | -0.230949397 | 2.42279E-06 | 0.000300952 |
| TPRG1L | 1p36.32 | -0.230297247 | 2.59094E-06 | 0.000314038 |
| CCDC178 | 18q12.1 | -0.229981753 | 2.67624E-06 | 0.000320492 |
| EEF1A1 | 6q13 | -0.229145958 | 2.9154E-06 | 0.000340965 |
| VPS36 | 13q14.3 | -0.228794175 | 3.02205E-06 | 0.000349584 |
| PPRC1 | 10q24.32 | -0.228202098 | 3.21002E-06 | 0.000364376 |
| WNT9A | 1q42.13 | -0.227516437 | 3.44166E-06 | 0.000382388 |
| C22ORF23 | 22q13.1 | -0.226529761 | 3.8032E-06 | 0.000414205 |
| TRIM7 | 5q35.3 | -0.226509795 | 3.81088E-06 | 0.000414205 |
| PPL | 16p13.3 | -0.225437838 | 4.24553E-06 | 0.000451629 |
| FAM83C | 20q11.22 | -0.223340238 | 5.2367E-06 | 0.000531618 |
| IMPDH2 | 3p21.31 | -0.223274488 | 5.27108E-06 | 0.000532406 |
| SLC38A9 | 5q11.2 | -0.223186145 | 5.31762E-06 | 0.000534407 |
| SLC12A9 | 7q22.1 | -0.222978362 | 5.42863E-06 | 0.000542836 |
| AGAP3 | 7q36.1 | -0.222435051 | 5.72943E-06 | 0.000563666 |
| LGI3 | 8p21.3 | -0.222399471 | 5.74968E-06 | 0.000563666 |
| WNT10A | 2q35 | -0.222309819 | 5.80099E-06 | 0.000565922 |
| DBNDD2 | 20q13.12 | -0.222091572 | 5.92773E-06 | 0.000575479 |
| SH2D3A | 19p13.3 | -0.221902341 | 6.03976E-06 | 0.000581011 |
| SPRYD7 | 13q14.2 | -0.221692261 | 6.16649E-06 | 0.000587256 |
| RPS23 | 5q14.2 | -0.22079593 | 6.73626E-06 | 0.000623697 |
| SNAI2 | 8q11.21 | -0.2205597 | 6.89458E-06 | 0.000632499 |
| TYSND1 | 10q22.1 | -0.219744469 | 7.46862E-06 | 0.000675859 |
| IL17RE | 3p25.3 | -0.219488097 | 7.65836E-06 | 0.000683913 |
| EEF1B2 | 2q33.3 | -0.215779624 | 1.09722E-05 | 0.00089962 |
| FSTL3 | 19p13.3 | -0.21562944 | 1.11317E-05 | 0.00090131 |
| BTRC | 10q24.32 | -0.214781522 | 1.20744E-05 | 0.000959077 |
| CTBP2 | 10q26.13 | -0.214772334 | 1.2085E-05 | 0.000959077 |
| MCTP2 | 15q26.2 | -0.214697949 | 1.21713E-05 | 0.000962108 |
| CBY2 | 13q14.13 | -0.214252494 | 1.27004E-05 | 0.000990417 |
| ADAMTSL5 | 19p13.3 | -0.21310194 | 1.41702E-05 | 0.001077526 |
| RPL12 | 9q33.3 | -0.21256764 | 1.49064E-05 | 0.001108227 |
| RHOD | 11q13.2 | -0.211720958 | 1.61479E-05 | 0.001182939 |
| DHRS12 | 13q14.3 | -0.210583451 | 1.79711E-05 | 0.001277318 |
| SBDS | 7q11.21 | -0.210093499 | 1.88152E-05 | 0.001298137 |
| HIF1AN | 10q24.31 | -0.208925071 | 2.09826E-05 | 0.001391182 |
| NBEAL2 | 3p21.31 | -0.208858814 | 2.11123E-05 | 0.001391182 |
| PKP3 | 11p15.5 | -0.208848919 | 2.11317E-05 | 0.001391182 |
| FLJ44635 | Xq13.1 | -0.20811674 | 2.26183E-05 | 0.001468649 |
| RPL8 | 8q24.3 | -0.207906473 | 2.30632E-05 | 0.001492691 |
| CCND1 | 11q13.3 | -0.207837742 | 2.32105E-05 | 0.001497374 |
| RELL2 | 5q31.3 | -0.207039314 | 2.49873E-05 | 0.001591468 |
| CCDC69 | 5q33.1 | -0.206833633 | 2.54655E-05 | 0.001601523 |
| HSPB1 | 7q11.23 | -0.20648291 | 2.63009E-05 | 0.001643726 |
| MROH6 | 8q24.3 | -0.206304104 | 2.67368E-05 | 0.001654012 |
| GOPC | 6q22.1 | -0.20621629 | 2.69534E-05 | 0.001658585 |
| MMRN1 | 4q22.1 | -0.206140769 | 2.71409E-05 | 0.001665004 |
| LIAS | 4p14 | -0.206028473 | 2.74221E-05 | 0.00167711 |
| LRRK1 | 15q26.3 | -0.204774882 | 3.07544E-05 | 0.00183599 |
| DIS3 | 13q21.33 | -0.204714809 | 3.09233E-05 | 0.001840581 |
| IDI2 | 10p15.3 | -0.204586181 | 3.1288E-05 | 0.001856762 |
| IGIP | 5q31.3 | -0.204235732 | 3.23024E-05 | 0.00190565 |
| INS-IGF2 | 11p15.5 | -0.204026681 | 3.29222E-05 | 0.001932047 |
| VWA8 | 13q14.11 | -0.204019724 | 3.2943E-05 | 0.001932047 |
| MAP3K7CL | 21q21.3 | -0.203416339 | 3.4797E-05 | 0.002028876 |
| SCEL | 13q22.3 | -0.203149532 | 3.56478E-05 | 0.002060465 |
| MGAM | 7q34 | -0.202819941 | 3.67261E-05 | 0.00208969 |
| DNAAF9 | 20p13 | -0.202803591 | 3.67804E-05 | 0.00208969 |
| NDFIP2 | 13q31.1 | -0.201624032 | 4.09033E-05 | 0.002258836 |
| SNHG5 | 6q14.3 | -0.200814807 | 4.39805E-05 | 0.002370799 |
| KCNK6 | 19q13.2 | -0.200698194 | 4.44416E-05 | 0.002389213 |
| RXRA | 9q34.2 | -0.20021213 | 4.6413E-05 | 0.002463155 |
| EPB41L4A-AS1 | 5q22.1 | -0.19994763 | 4.75203E-05 | 0.002495438 |
| KRT5 | 12q13.13 | -0.19994286 | 4.75405E-05 | 0.002495438 |
| CAPN3 | 15q15.1 | -0.19964903 | 4.88002E-05 | 0.00253766 |
| KRT15 | 17q21.2 | -0.199637015 | 4.88524E-05 | 0.00253766 |
| HSPB1P1 | 9q21.13 | -0.199233463 | 5.06358E-05 | 0.002596579 |
| TM9SF3 | 10q24.1 | -0.19902621 | 5.15755E-05 | 0.002624575 |
| EVPL | 17q25.1 | -0.198632022 | 5.34083E-05 | 0.002680657 |
| RNF170 | 8p11.21 | -0.198616474 | 5.34818E-05 | 0.002680657 |
| GSTK1 | 7q34 | -0.198220166 | 5.53888E-05 | 0.002762393 |
| TNNT2 | 1q32.1 | -0.198094672 | 5.60059E-05 | 0.002779309 |
| LTBP4 | 19q13.2 | -0.198033409 | 5.63095E-05 | 0.002782745 |
| PLEKHA1 | 10q26.13 | -0.198024574 | 5.63534E-05 | 0.002782745 |
| HOXA1 | 7p15.2 | -0.197837817 | 5.72892E-05 | 0.002808156 |
| LINC00924 | 15q26.2 | -0.197483685 | 5.91042E-05 | 0.002875974 |
| MAFK | 7p22.3 | -0.197436562 | 5.93498E-05 | 0.002876548 |
| RPL13 | 16q24.3 | -0.197339914 | 5.98564E-05 | 0.002884503 |
| PTK6 | 20q13.33 | -0.196646243 | 6.36144E-05 | 0.00302191 |
| LSP1 | 11p15.5 | -0.196251525 | 6.5851E-05 | 0.003091441 |
| BAIAP2 | 17q25.3 | -0.196036498 | 6.71004E-05 | 0.003123479 |
| PHACTR3 | 20q13.32-q13.33 | -0.196026646 | 6.71582E-05 | 0.003123479 |
| FGFR3 | 4p16.3 | -0.195852744 | 6.81858E-05 | 0.0031493 |
| DSC3 | 18q12.1 | -0.195724781 | 6.89513E-05 | 0.003176112 |
| MCC | 5q22.2 | -0.195622698 | 6.95679E-05 | 0.003176112 |
| GNRHR | 4q13.2 | -0.195500459 | 7.0313E-05 | 0.003195886 |
| CYB5R1 | 1q32.1 | -0.19510165 | 7.27966E-05 | 0.003293801 |
| RPS12 | 6q23.2 | -0.194968428 | 7.36446E-05 | 0.003324645 |
| SPRYD3 | 12q13.13 | -0.194797925 | 7.47433E-05 | 0.003359083 |
| KIF1C | 17p13.2 | -0.194770539 | 7.49213E-05 | 0.003359529 |
| TRIM13 | 13q14.2 | -0.194724247 | 7.52229E-05 | 0.00336551 |
| MIR205HG | 1q32.2 | -0.19462207 | 7.58928E-05 | 0.003387901 |
| PDGFA | 7p22.3 | -0.1942191 | 7.85898E-05 | 0.003484961 |
| MFSD3 | 8q24.3 | -0.193990115 | 8.01623E-05 | 0.003524643 |
| C1ORF116 | 1q32.1 | -0.193976863 | 8.02542E-05 | 0.003524643 |
| SH3PXD2A | 10q24.33 | -0.193844702 | 8.11763E-05 | 0.003544638 |
| PLD2 | 17p13.2 | -0.193803887 | 8.1463E-05 | 0.00354941 |
| RIOK2 | 5q15 | -0.193708653 | 8.21359E-05 | 0.003570945 |
| MBLAC2 | 5q14.3 | -0.193225062 | 8.56339E-05 | 0.00369093 |
| QARS1 | 3p21.31 | -0.193080003 | 8.67102E-05 | 0.003729284 |
| SNX33 | 15q24.2 | -0.192969574 | 8.75381E-05 | 0.003746788 |
| GLUD1 | 10q23.2 | -0.192956322 | 8.76379E-05 | 0.003746788 |
| SLC4A11 | 20p13 | -0.192950845 | 8.76792E-05 | 0.003746788 |
| NAALADL1 | 11q13.1 | -0.192502246 | 9.11247E-05 | 0.003861023 |
| KLHL30 | 2q37.3 | -0.191250233 | 0.000101427 | 0.004234743 |
| TRIM41 | 5q35.3 | -0.190875662 | 0.000104716 | 0.004344377 |
| DSG3 | 18q12.1 | -0.190852603 | 0.000104922 | 0.004344377 |
| CRYBG3 | 3q11.2 | -0.189770841 | 0.000115011 | 0.004606899 |
| HERC4 | 10q21.3 | -0.189588501 | 0.000116799 | 0.004643872 |
| C19ORF33 | 19q13.2 | -0.189556344 | 0.000117117 | 0.00464466 |
| THSD1 | 13q14.3 | -0.189398739 | 0.000118688 | 0.004690975 |
| PLCD3 | 17q21.31 | -0.189296615 | 0.000119716 | 0.004712983 |
| TICAM1 | 19p13.3 | -0.188825038 | 0.000124573 | 0.004821727 |
| RPLP1 | 15q23 | -0.188772032 | 0.00012513 | 0.004821727 |
| FNDC3A | 13q14.2 | -0.188549231 | 0.000127499 | 0.00488955 |
| ETNK2 | 1q32.1 | -0.188537569 | 0.000127624 | 0.00488955 |
| PLEKHG5 | 1p36.31 | -0.18836318 | 0.000129509 | 0.004933411 |
| KIF9 | 3p21.31 | -0.188195504 | 0.000131345 | 0.004991203 |
| NOP16 | 5q35.2 | -0.188134724 | 0.000132017 | 0.005000405 |
| TFAP2A | 6p24.3 | -0.187707143 | 0.000136836 | 0.005173119 |
| EEF2 | 19p13.3 | -0.18738628 | 0.00014056 | 0.005283932 |
| AHNAK2 | 14q32.33 | -0.187136092 | 0.000143529 | 0.005365299 |
| ABT1 | 6p22.2 | -0.186895092 | 0.000146445 | 0.00539016 |
| CSRP1 | 1q32.1 | -0.18688078 | 0.00014662 | 0.00539016 |
| B4GALT2 | 1p34.1 | -0.186704447 | 0.000148791 | 0.005449934 |
| TMEM220 | 17p13.1 | -0.186579626 | 0.000150346 | 0.005486791 |
| CTDSPL | 3p22.2 | -0.186335702 | 0.000153428 | 0.005538654 |
| METTL8 | 2q31.1 | -0.18613587 | 0.000155998 | 0.005611158 |
| ADAT2 | 6q24.2 | -0.185991348 | 0.000157881 | 0.005665854 |
| FAM174A | 5q21.1 | -0.185954236 | 0.000158369 | 0.005665854 |
| KBTBD6 | 13q14.11 | -0.185647862 | 0.000162444 | 0.005729777 |
| SLC12A4 | 16q22.1 | -0.185647508 | 0.000162449 | 0.005729777 |
| NUTM2A-AS1 | 10q23.2 | -0.185608284 | 0.000162977 | 0.005729777 |
| EEF1D | 8q24.3 | -0.185457217 | 0.000165029 | 0.005790204 |
| RACK1 | 5q35.3 | -0.185090239 | 0.000170114 | 0.005927038 |
| ATXN7L1 | 7q22.3 | -0.184580322 | 0.000177425 | 0.006070451 |
| C8ORF82 | 8q24.3 | -0.18447908 | 0.000178911 | 0.006105545 |
| ALPP | 2q37.1 | -0.184459018 | 0.000179207 | 0.006105545 |
| RRP12 | 10q24.1 | -0.183522146 | 0.000193544 | 0.006408428 |
| C6ORF132 | 6p21.1 | -0.183418431 | 0.000195196 | 0.006441784 |
| SRRM3 | 7q11.23 | -0.183391398 | 0.000195628 | 0.006445426 |
| MAPK11 | 22q13.33 | -0.183366839 | 0.000196022 | 0.00644778 |
| ESPNL | 2q37.3 | -0.183263054 | 0.000197695 | 0.006470866 |
| PI15 | 8q21.13 | -0.183238578 | 0.000198091 | 0.006473245 |
| ALPG | 2q37.1 | -0.182992255 | 0.000202122 | 0.006572743 |
| MRPS31 | 13q14.11 | -0.182898796 | 0.000203671 | 0.006598873 |
| PPP1R14B | 11q13.1 | -0.18278448 | 0.000205581 | 0.006631328 |
| TTLL12 | 22q13.2 | -0.182447636 | 0.000211308 | 0.006739949 |
| LIPN | 10q23.31 | -0.182127033 | 0.000216897 | 0.006885265 |
| PDLIM1 | 10q23.33 | -0.18204381 | 0.00021837 | 0.006921044 |
| RPL13AP20 | 12p13.1 | -0.18170899 | 0.000224392 | 0.007100652 |
| ANKS6 | 9q22.33 | -0.181646089 | 0.00022554 | 0.007103277 |
| SERPINB5 | 18q21.33 | -0.181605548 | 0.000226283 | 0.007115476 |
| EML3 | 11q12.3 | -0.18138636 | 0.000230341 | 0.007220364 |
| MT1E | 16q13 | -0.181258616 | 0.000232737 | 0.007280992 |
| COQ4 | 9q34.11 | -0.181225222 | 0.000233367 | 0.007280992 |
| HPS6 | 10q24.32 | -0.180770254 | 0.000242114 | 0.007495999 |
| IL33 | 9p24.1 | -0.1804948 | 0.000247557 | 0.007611143 |
| PLAG1 | 8q12.1 | -0.180484905 | 0.000247755 | 0.007611143 |
| DLEU1 | 13q14.2-q14.3 | -0.18034603 | 0.000250545 | 0.007662416 |
| RPS13 | 11p15.1 | -0.18028737 | 0.000251732 | 0.007676334 |
| KLHL17 | 1p36.33 | -0.180265284 | 0.00025218 | 0.007676334 |
| OGFOD1 | 16q13 | -0.179881521 | 0.00026009 | 0.007857614 |

Supplementary Figure 1: (A) PDK3 co-upregulated genes and (B) PDK3 negative correlated genes in terms of biological processes.


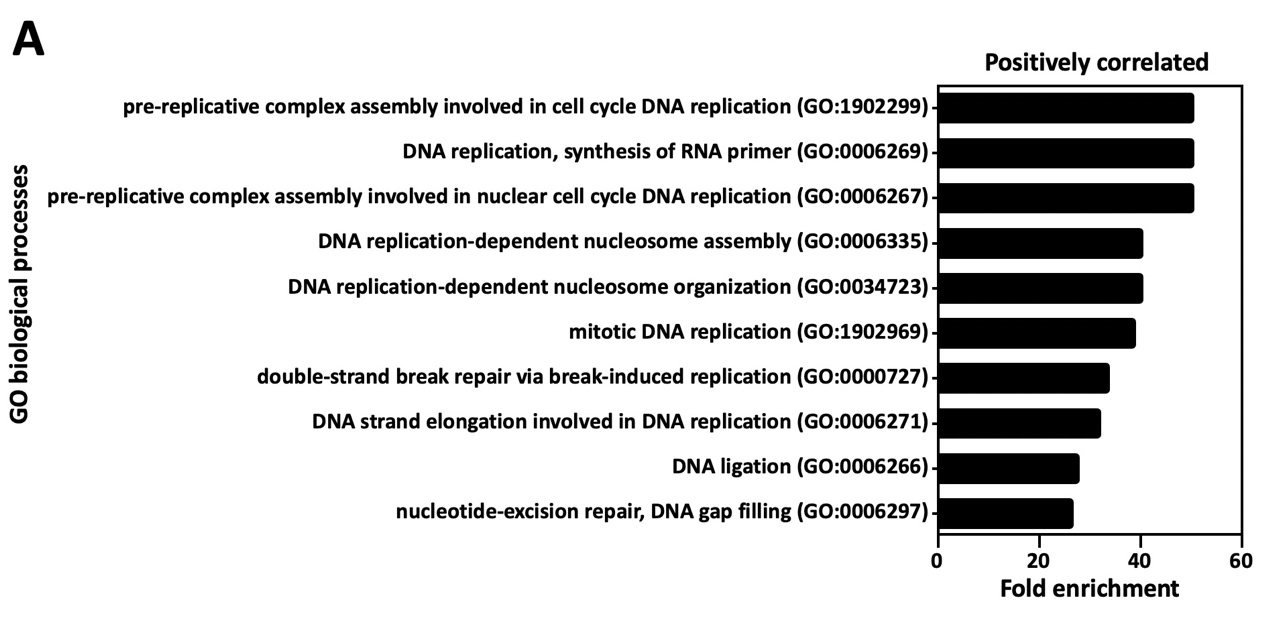


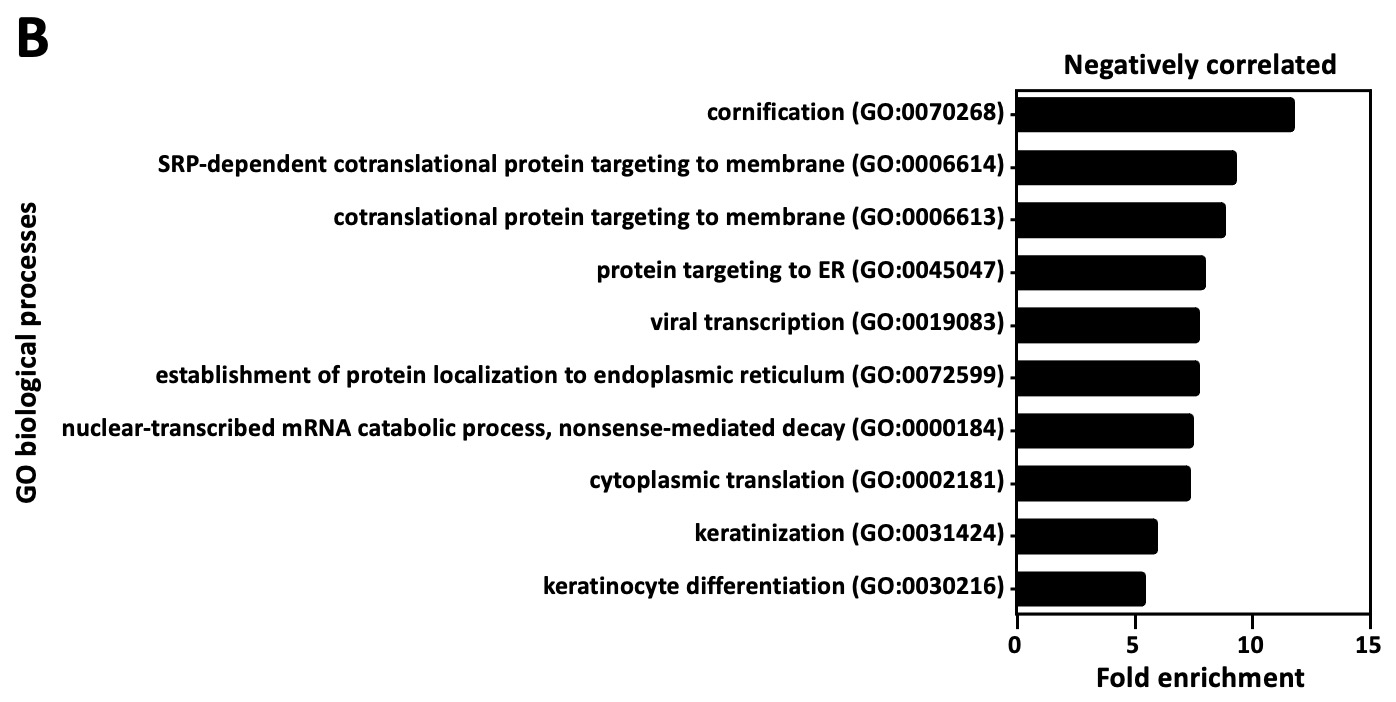


Supplementary Figure 2: (A) PDK3 co-upregulated genes and (B) PDK3 negative correlated genes in terms of cellular component.


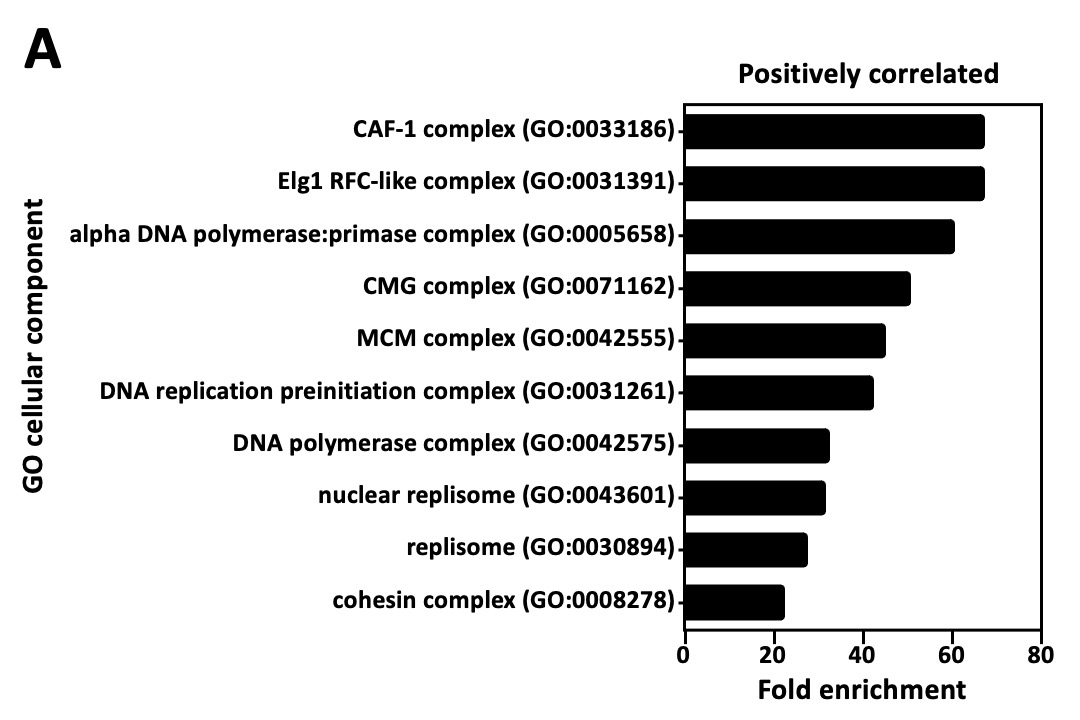


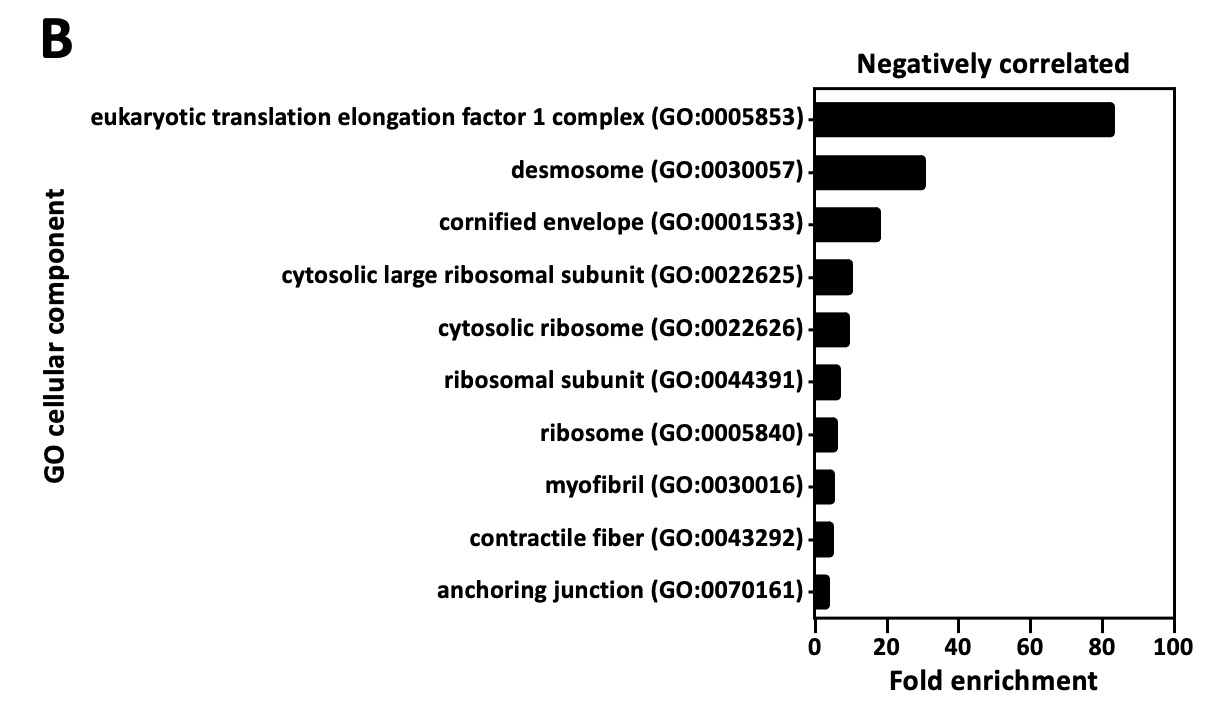


Supplementary Figure 3: In patients with upper tract urothelial carcinoma (A,B, respectively) and urinary bladder urothelial carcinoma (C,D, respectively), Kaplan–Meier plots reveal that PDK1 overexpression had substantial prognostic impacts on disease-specific survival and metastasis-free survival.

1.
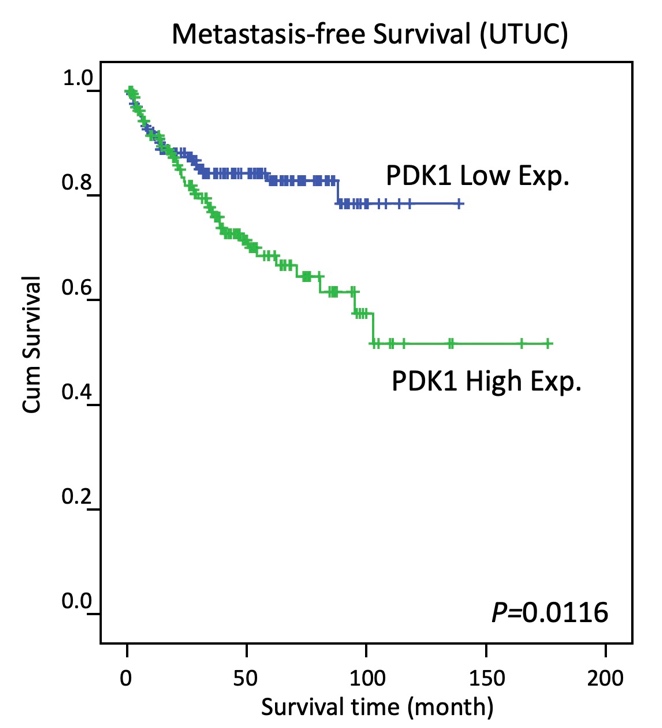

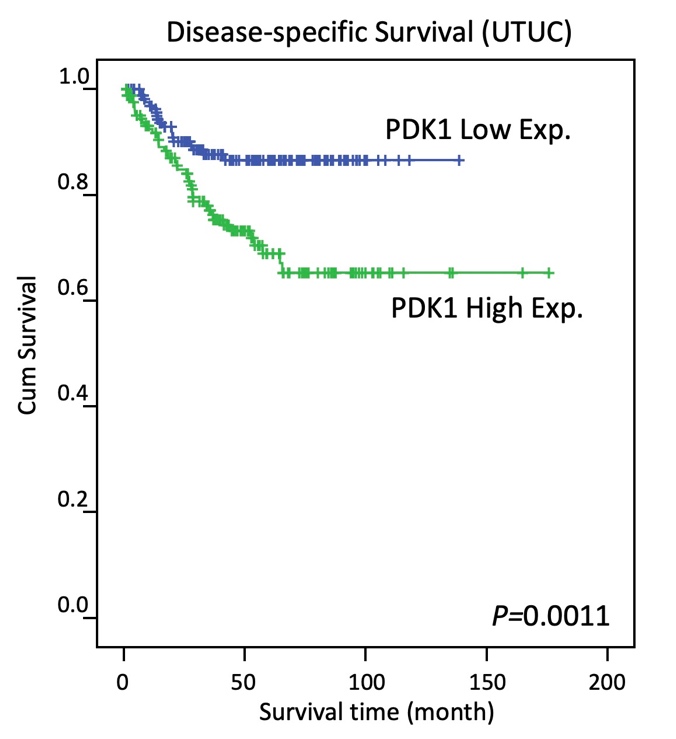
 **(B)**


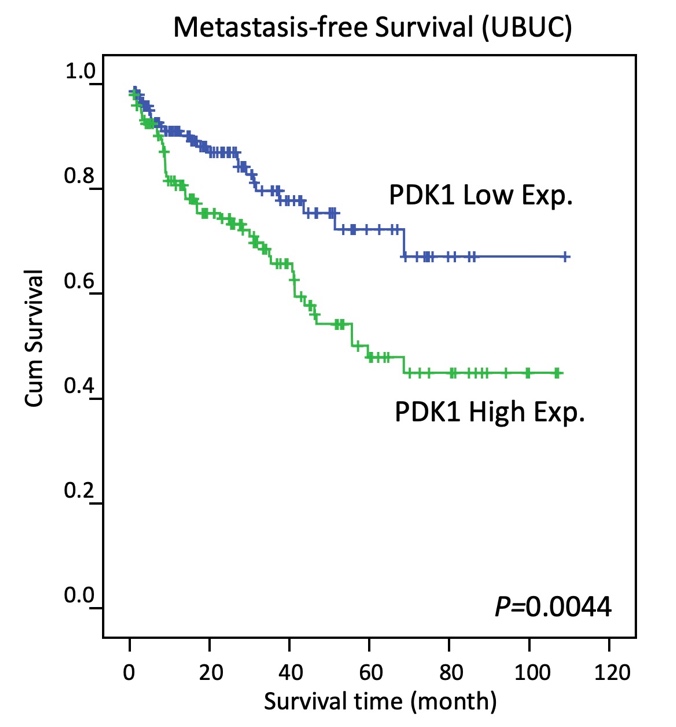
**(C) (D)**


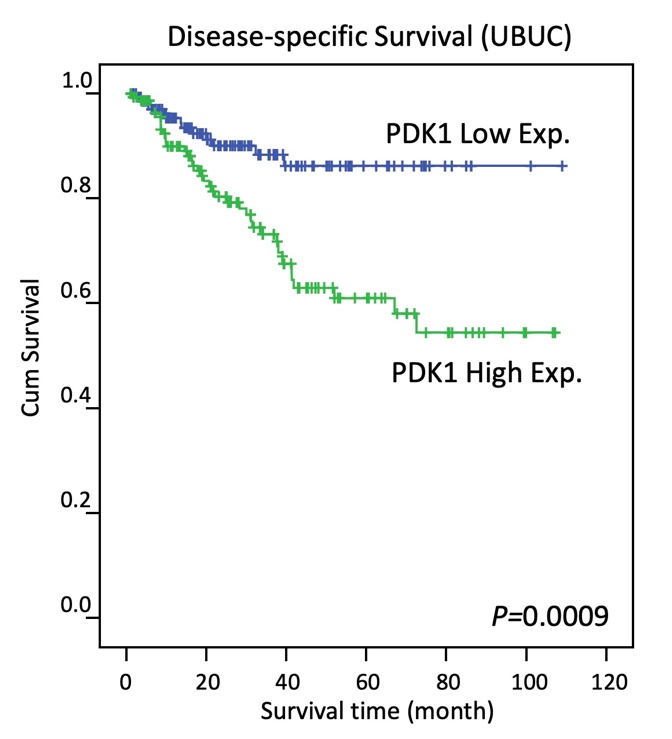

Supplement: Supplementary file 1 [file DataSheet_1.docx]
